# Surface Plasmon Resonance-Based Biodetection Systems: Principles, Progress and Applications—A Comprehensive Review

**DOI:** 10.3390/bios15010035

**Published:** 2025-01-09

**Authors:** Muhammad A. Butt

**Affiliations:** Institute of Microelectronics and Optoelectronics, Warsaw University of Technology, Koszykowa 75, 00-662 Warsaw, Poland; ali.butt@pw.edu.pl

**Keywords:** Surface Plasmon Resonance, plasmonics, biodetection systems, food safety, environmental monitoring, drug delivery

## Abstract

Surface Plasmon Resonance (SPR)-based biodetection systems have emerged as powerful tools for real-time, label-free biomolecular interaction analysis, revolutionizing fields such as diagnostics, drug discovery, and environmental monitoring. This review highlights the foundational principles of SPR, focusing on the interplay of evanescent waves and surface plasmons that underpin its high sensitivity and specificity. Recent advancements in SPR technology, including enhancements in sensor chip materials, integration with nanostructures, and coupling with complementary detection techniques, are discussed to showcase their role in improving analytical performance. The paper also explores diverse applications of SPR biodetection systems, ranging from pathogen detection and cancer biomarker identification to food safety monitoring and environmental toxin analysis. By providing a comprehensive overview of technological progress and emerging trends, this review underscores the transformative potential of SPR-based biodetection systems in addressing critical scientific and societal challenges. Future directions and challenges, including miniaturization, cost reduction, and expanding multiplexing capabilities, are also presented to guide ongoing research and development in this rapidly evolving field.

## 1. Introduction

Surface Plasmon Resonance (SPR) is an optical effect that occurs when polarized light interacts with electrons at the interface between a metal and a dielectric material, resulting in the generation of surface plasmons (SPs)—coherent oscillations of the electrons [1,2]. This interaction leads to the propagation of an electromagnetic wave along the metal–dielectric interface [3,4]. SPR is highly sensitive to variations in the refractive index (RI) near the metal surface, making it a valuable method for detecting molecular interactions in real time without the need for labels [5]. In SPR experiments, polarized light is directed at a thin metal film, often gold (Au), under conditions that meet the resonance criteria. At a specific angle or wavelength, the energy of the incident photons aligns with the energy needed to excite the SPs, causing a drop in the intensity of the reflected light [6]. This resonance is highly sensitive to changes in the RI of the surrounding medium. When a molecule attaches to a ligand that is fixed on the metal surface, the local refractive index changes, causing a shift in the resonance angle or wavelength [7]. By observing these shifts, SPR allows for the precise measurement of biomolecular interactions, including binding affinities, kinetics, and concentrations [8,9].

SPR is renowned for its exceptional sensitivity to changes in RI at the nanoscale, allowing it to identify minute quantities of analyte [10]. The technology can measure binding events in real-time with limit of detection (LOD) often in the picomolar (pM) to nanomolar (nM) range. This high sensitivity stems from the exponential decay of the evanescent field generated at the metal surface, which interacts specifically with molecules close to the surface (typically within 200 nm) [11]. This localized sensitivity is ideal for studying interactions involving biomolecules such as proteins, nucleic acids, lipids, and small molecules [12]. SPR’s specificity is enhanced using functionalized surfaces tailored for the selective capture of target analytes. Immobilization strategies, such as covalent coupling or affinity-based interactions, ensure that the response is predominantly due to specific binding events rather than nonspecific adsorption [13]. Moreover, the ability to measure kinetic parameters (e.g., association and dissociation rates) provides an additional layer of specificity, as these rates are characteristic of molecular interplays [7].

SPR technology is highly versatile, accommodating a wide range of applications across diverse scientific and industrial fields [2,14,15]. Its adaptability stems from the availability of various surface chemistries and experimental configurations, enabling the study of interplays between different classes of molecules [16]. SPR can be used to investigate protein–protein, protein–DNA, protein–lipid, and receptor–ligand interactions, among others [12,17]. In addition, SPR systems can be integrated with complementary analytical techniques, such as mass spectrometry or fluorescence, to provide richer data [18]. The technology’s non-invasive, label-free nature also makes it suitable for studying live cells, vesicles, or other complex biological systems [19].

The roots of SPR technology trace back to the early 20th century when theoretical foundations for SPs were first laid [20]. In 1902, Wood’s anomaly was observed, which later paved the way for understanding diffraction effects on metallic surfaces [21]. In 1968, Otto and Kretschmann independently demonstrated the excitation of SPs via the prism coupling method, a pivotal moment that formalized SPR as a distinct optical phenomenon [22,23]. These setups—commonly known as Otto and Kretschmann configurations—remain fundamental to SPR experimental designs. The 1980s marked the transition of SPR from a purely physical phenomenon to an applied analytical technique. During this period, researchers recognized the potential use of SPR in monitoring changes at the metal–dielectric interface. By the late 1980s, commercial SPR biodetection systems were developed, primarily targeting the pharmaceutical and biomedical industries. Companies like Biacore introduced systems capable of real-time, label-free biomolecular interplay analysis, a breakthrough in bioanalytical science. These early devices relied on advancements in optics and computational power, enabling the precise detection of resonance angle shifts [24].

SPR-based sensors and Raman spectroscopy are prominent biomolecular detection techniques, each with unique strengths and applications. Raman spectroscopy provides molecular fingerprints based on the inelastic scattering of light, offering detailed structural and chemical information about biomolecules [25]. While SPR is more suited for dynamic monitoring and quantifying interactions, Raman spectroscopy excels in identifying molecular compositions and studying conformational changes. However, Raman signals are often weak and require enhancement techniques, such as Surface-Enhanced Raman Spectroscopy (SERS), to improve sensitivity [26]. Direct comparison highlights that SPR offers higher throughput and specificity for interaction studies, whereas Raman spectroscopy provides richer molecular characterization, making their applications complementary in advanced biosensing platforms [27].

Modern SPR technology has significantly evolved, incorporating nanotechnology, microfluidics, and multiplexing capabilities [28,29]. High-throughput SPR systems now enable the simultaneous analysis of multiple interplays, drastically improving efficiency in drug discovery and diagnostics. Integration with complementary methods like mass spectrometry and the advent of localized SPR (LSPR), utilizing nanoparticles, have further expanded the scope of applications [30,31]. These innovations have transformed SPR into a versatile tool used not only in fundamental research, but also in fields like environmental monitoring, food safety, and material science [32]. The advent of SPR technology has profoundly influenced biosensing research and industrial applications, establishing it as a cornerstone of modern analytical methods. In biosensing, SPR has enabled the real-time, label-free detection of biomolecular interplays, a capability that revolutionized the understanding of biological processes. Unlike traditional methods requiring labeled molecules, SPR’s reliance on changes in RI provides a non-invasive approach, preserving the integrity of biological samples [33]. This has made SPR indispensable in studying complex systems such as protein–protein interplays, antibody–antigen binding, and receptor–ligand dynamics.

Industrially, SPR has had a transformative impact, particularly in pharmaceuticals and diagnostics [34]. Drug discovery pipelines have been streamlined by SPR’s ability to provide kinetic and affinity data, aiding in the identification of promising therapeutic candidates. This capability reduces the time and cost associated with traditional high-throughput screening. SPR biodetection systems are also pivotal in diagnostics, enabling the detection of biomarkers for diseases like cancer, infectious diseases, and autoimmune disorders. For instance, SPR-based systems are extensively employed for the rapid and accurate detection of viral proteins, such as in HIV-1 or SARS-CoV-2 testing [35,36]. Beyond biomedicine, SPR has extended its influence into fields like environmental science and food safety [37]. SPR-based sensors monitor pollutants, pesticides, and other hazardous substances with high precision [38]. In the food industry, SPR is employed to identify contaminants and ensure quality control. Additionally, SPR’s adaptability has catalyzed advancements in materials science, where it aids in characterizing thin films, coatings, and nanostructures [39].

Ensuring the quality and consistency of SPR biosensor chips is critical for achieving reliable detection results. This can be accomplished by standardizing the fabrication processes, including the deposition of the gold film and surface functionalization steps [40,41,42]. High-quality gold films with uniform thickness and smooth surfaces are essential, as surface roughness can significantly affect plasmon resonance and sensor sensitivity. Reproducible functionalization protocols, such as consistent chemical treatments for immobilizing biomolecules, help reduce variability between chips [43]. To minimize the impacts of chip-to-chip differences on detection results, thorough calibration and quality control procedures should be implemented. Utilizing reference samples and internal standards during each experiment allows for the normalization of results across different chips. Additionally, employing well-characterized, commercially produced chips with stringent quality assurance can further enhance consistency. By addressing these factors, researchers can improve the reproducibility and reliability of SPR biosensor-based assays [44].

The purpose of this review is to synthesize key advancements in SPR technology, highlighting its evolution and expanding applications across scientific and industrial domains. By consolidating significant milestones, from its foundational principles to modern innovations, the review aims to provide a comprehensive understanding of SPR’s trajectory. Furthermore, it seeks to identify emerging trends and novel applications, offering insights into future directions in biosensing, diagnostics, and beyond. This synthesis serves as a valuable resource for researchers and practitioners, fostering continued innovation and interdisciplinary collaboration in SPR technology.

## 2. Working Principle, Configurations and Detection Methods of SPR-Based Biodetection Systems

In this section, the fundamental aspects of SPR-based biodetection systems, focusing on their working principle, various configurations, and the diverse detection methods employed to enhance their performance, are discussed. The working principle of SPR involves the excitation of SPs at the interface of a thin metal layer and a dielectric medium, driven by changes in the RI caused by biomolecular interplays. Configurations such as the Kretschmann prism setup, optical waveguides, and nanoparticle-enhanced systems are explored to highlight how different designs cater to specific application needs. Additionally, detection methods ranging from the direct monitoring of RI changes to advanced signal amplification techniques are discussed, showcasing the versatility and adaptability of SPR-based biosensors in various scientific and industrial applications.

### 2.1. Working Principle of SPR-Based Biodetection Systems

SPs, collective oscillations of free electrons, are excited at the metal–dielectric interface, such as between a metal and a glass prism or sensing medium like water. When light is directed at this boundary, part of its energy transfers to the SPs, creating resonance when the photon momentum aligns with that of the SPs. Because SPs are confined to the interface, their behavior is highly sensitive to changes in the RI of the surrounding medium [14]. This sensitivity underpins SPR biodetection systems, allowing the detection of subtle changes like biomolecular interactions. The coupling of light and SPs is greatly affected by the light’s polarization. For SPR to be initiated, the incident light must be p-polarized (TM mode), meaning its electric field component must be perpendicular to the metal surface. This polarization allows the electromagnetic wave to interact effectively with the charge density oscillations at the metal interface.

For classical metals like Au and Ag, their electronic structures—dominated by free electron behavior described by the Drude model—result in distinct optical responses [45,46]. Ag, with its lower intrinsic losses and a sharper plasmonic resonance, exhibits stronger field enhancement and sharper SPR peaks compared to Au, which has higher intrinsic losses due to interband transitions near the visible spectrum. These interband transitions contribute to the broader and less intense SPR in Au. Novel materials, such as doped semiconductors, 2D materials (e.g., graphene), or transition metal nitrides, introduce additional complexities due to their unconventional electronic structures, including tunable bandgaps and anisotropic dielectric responses [47]. Such features enable the tailoring of the plasmonic resonance to specific wavelengths and applications. These differences in electronic structures determine the material’s permittivity, impacting the resonance frequency, field confinement, and propagation length of SPs. The interplay between optical properties and electronic structure highlights the potential of novel materials to surpass the limitations of traditional metals, paving the way for advanced plasmonic applications in sensing, imaging, and photonic devices [48].

**Figure 1:**
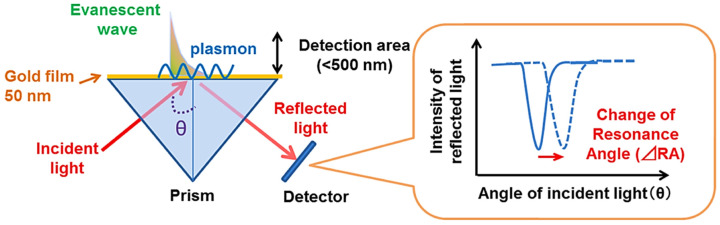
SPR sensors operate by detecting changes in RI within a detection area of less than 500 nm, which are observed as variations in the resonance angle [50].

Resonance in SPR systems occurs only under specific conditions. One key factor is the angle of incidence of the light [49]. In the Kretschmann configuration, light enters through a prism and reflects off a thin metallic layer. By adjusting the angle of incidence, a resonance point is reached, marked by a distinct decrease in reflected light intensity (Figure 1) [50]. This dip signifies energy transfer to surface plasmons (SPs) and is influenced by the refractive index (RI) of the material adjacent to the metal [51]. The wavelength of the incident light is another critical factor. At a constant angle, resonance occurs only at specific wavelengths, determined by the metal’s properties and the refractive indices of the materials involved [52]. SPR systems generally function by either varying the angle at a constant wavelength or adjusting the wavelength at a fixed angle to determine the resonance condition [53]. The RI of the medium near the metal surface is a key factor in SPR. Any changes in this RI, such as those caused by the biomolecules binding to a functionalized sensor surface, modify the resonance condition. These shifts can be detected by observing changes in the resonance angle, wavelength, or reflected light intensity. Due to its exceptional sensitivity to RI variations, SPR provides an ideal platform for the real-time, label-free detection and analysis of biomolecular interactions [54].

### 2.2. SPR Configurations and Detection Methods

This section provides a detailed discussion on various SPR configurations and the associated detection methods, highlighting their unique characteristics and applications.

#### 2.2.1. Prism Coupling (Kretschmann and Otto Configurations)

**Figure 2:**
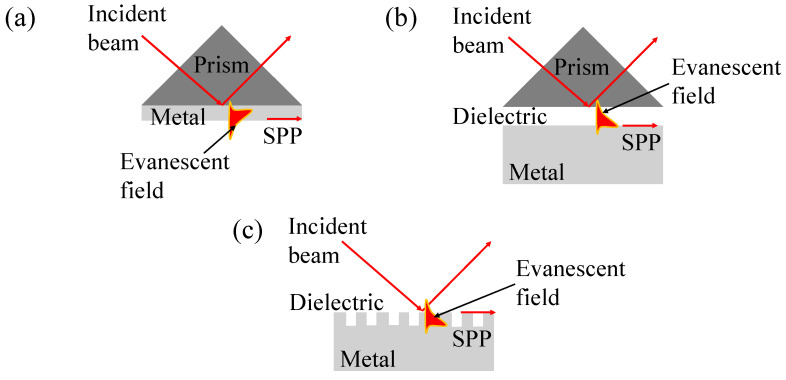
SPR, (**a**) Kretschmann configuration, (**b**) Otto configuration, (**c**) diffraction grating. SPP stands for surface plasmon polariton. In Kretschmann configuration, analytes are introduced in a sample solution that comes into direct contact with the metal film. In Otto configuration, analytes are placed in a sample medium that is separated from the prism by a thin air gap or dielectric spacer, whereas in diffraction grating configuration, analytes are introduced in a sample medium that is in contact with the surface of the diffraction grating, which is coated with a thin metal layer.

Prism coupling is the most widely utilized configuration for exciting SPs, primarily because it provides an efficient and controlled mechanism for coupling light to the plasmonic surface. In the Kretschmann configuration, a thin metallic film, typically gold or silver, is applied to a high-refractive-index prism (Figure 2a). Light passes through the prism, causing total internal reflection at the metal–dielectric interface. When the incident photon momentum matches that of the SPs, resonance occurs, resulting in a sharp dip in reflected light intensity, indicating energy transfer to the SPs.

In the Otto configuration, an air gap or another dielectric medium is placed between the prism and the metal surface (Figure 2b). This separation ensures that evanescent waves, generated by total internal reflection within the prism, excite the SPs. While less commonly used than the Kretschmann configuration, the Otto setup allows greater flexibility in tuning the interplay by varying the gap width. However, its practical implementation is more challenging, as maintaining a uniform and precise air gap can be difficult. Both Kretschmann and Otto configurations are central to SPR-based biosensing. By tracking shifts in the resonance angle caused by changes in the RI of the medium adjacent to the metal surface, these configurations enable the sensitive, label-free detection of molecular interplays, making them ideal for applications in real-time biomolecular analysis.

Diffraction grating-based SPR sensors are innovative optical devices that leverage the coupling of light to SPs—electron density waves at the interface of a metal and a dielectric medium—to identify changes in the RI of a surrounding medium [55,56]. Unlike traditional prism-based SPR sensors, these systems utilize a diffraction grating to diffract incident light into angles that facilitate the resonance excitation of SPs (Figure 2c). This approach enables compact sensor designs while maintaining high sensitivity and specificity. Diffraction grating SPR sensors are generally employed in biosensing applications, allowing for the real-time, label-free detection of biomolecular interplays [57,58]. They are particularly advantageous due to their compatibility with miniaturized and multiplexed sensor arrays, which is critical for high-throughput analysis in medical diagnostics, environmental monitoring, and food safety [59].

The adsorption and desorption rates of biomolecules play a crucial role in determining the dynamic changes observed in the resonance signal of SPR biosensors. Adsorption refers to the binding of biomolecules to the sensor surface, while desorption is the release of these molecules back into the solution [60]. These processes are governed by kinetic rates, which influence the accumulation of or reduction in biomolecules on the sensor interface. During adsorption, the increased molecular density near the sensor surface alters the refractive index, causing a shift in the SPR resonance angle or intensity, which is detected as a change in the signal. Conversely, desorption decreases the refractive index, leading to a signal decline. The balance between these rates determines the overall response curve, including its association and dissociation phases, providing critical information about the interaction kinetics, binding affinities, and stability of the biomolecular complexes. Understanding and optimizing these rates is essential for accurate and reliable biosensor performance [61].

Lee et al. developed a micromachined Otto configuration chip with air gaps of different sizes (1.86 μm, 2.42 μm, 3.01 μm, and 3.43 μm) and analyzed its resonance using a 980 nm laser [62]. To assess reflectance variation and its suitability for multi-gas detection, the air gap was precisely controlled with a piezoactuator. The results show that both the air-gap distance and incident light wavelength significantly impacted the SPR behavior. With a 977 nm light source, reflectance reached a minimum of 0.22 at a piezoactuator displacement of approximately 9.3 μm.

When selecting the most suitable SPR configuration for practical applications, such as the prism coupling Kretschmann and Otto configurations, it is crucial to consider the specific detection requirements and experimental conditions. The Kretschmann configuration is commonly preferred for its ease of implementation and strong sensitivity, making it ideal for detecting small molecules or low concentrations [63]. On the other hand, the Otto configuration, which requires a small gap between the prism and metal film, offers enhanced flexibility for samples with higher refractive indices, but is more challenging to set up. Key factors influencing the choice include the size of the target molecules, as smaller molecules may necessitate a configuration with higher sensitivity, and the concentration range, where robustness and stability in signal are critical. Additionally, the sample’s optical properties, such as refractive index and absorption characteristics, play a vital role in determining the compatibility of the configuration. By carefully aligning these parameters with the strengths of each SPR configuration, researchers can optimize their experimental outcomes [51].

#### 2.2.2. Optical Waveguides and Fiber-Optic SPR

Optical waveguides and fiber-optic SPR systems represent an evolution of traditional prism-based methods, offering enhanced miniaturization, flexibility, and the ability to integrate into portable devices [64,65,66]. In these configurations, a metallic layer is deposited along the length of an optical waveguide or fiber. Light propagating through the waveguide or fiber generates an evanescent field that interacts with the metallic layer, exciting SPs under resonance conditions [67]. The detection of SPR in these systems typically involves measuring changes in transmitted or reflected light intensity [68,69].

Fiber-optic SPR systems, in particular, are highly advantageous for applications requiring compact and flexible sensing platforms [67,70,71]. These systems can be designed for remote or in situ sensing, as the fibers can be easily deployed in complex or constrained environments [72]. Additionally, fiber-optic SPR sensors can be tailored to specific applications by functionalizing the metallic surface with biomolecular recognition elements, enabling the highly selective detection of target analytes. The versatility of fiber-optic SPR systems extends to diverse fields, including environmental monitoring, medical diagnostics, and chemical analysis [69].

A common approach to their fabrication involves chemically immobilizing Au nanoparticles (AuNPs) on the fiber’s end face, a method valued for its simplicity and adaptability. However, this process often suffers from poor reproducibility due to the numerous factors affecting AuNP binding. To address this, Calatayud-Sanchez et al. investigated the influence of parameters such as temperature, AuNP concentration, fiber core size, and immersion time on both the density and aggregation of AuNPs and their resulting resonance signal [73]. This approach involved the real-time monitoring of the LSPR (plasmonic) signal to precisely control the deposition of a specific AuNP density onto the fiber tip. The resulting sensors were tested for their ability to identify changes in the surrounding RI. The findings reveal that as the number of AuNPs on the sensor increased, the maximum Sp value changes decreased, while wavelength shifts became more pronounced. These results underscore the critical importance of optimizing the balance between sensor composition and performance [73].

**Figure 3:**
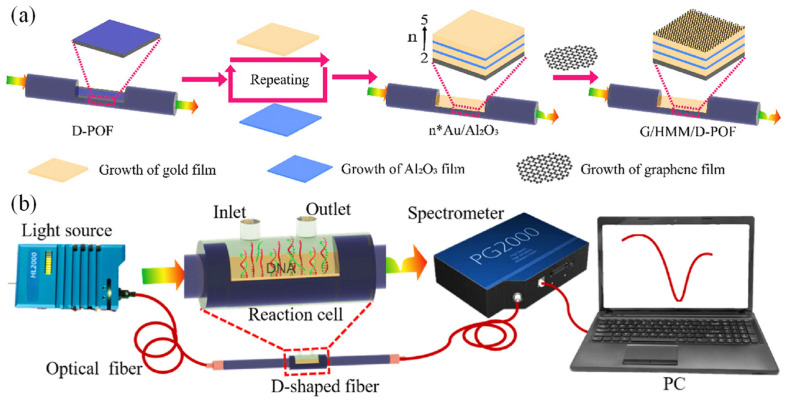
(**a**) Preparation process of G/HMM/D-POF, (**b**) schematic of an experimental setup based on G/HMM/D–POF sensor [71].

Li et al. developed an advanced optical fiber-based SPR biosensor tailored for the real-time analysis of DNA hybridization kinetics across various concentrations [71]. The biosensor incorporated a unique combination of components, including a 3D multilayer hyperbolic metamaterial (HMM) made of Au and Al_2_O_3_, a graphene layer, and a D-shaped plastic optical fiber (D-POF). The fabrication steps for constructing the G/HMM/D-POF structure are illustrated in Figure 3a. The composite HMMs were created by alternating layers of Au and Al_2_O_3_, with the Al_2_O_3_ acting as a spacer to divide the Au into multiple distinct layers (n-layer Au/Al_2_O_3_), where the number of layers (n) varied between 2 and 5. The total Au thickness in the HMM structure was set at 50 nm to match the optimal functional layer thickness of conventional single-layer Au SPR sensors, which typically ranges from 30 to 80 nm. Importantly, the overall thickness of the functional layers was kept below 80 nm. In the fabrication process, an Au layer was thermally deposited onto the surface of the POF at a rate of 0.7 Å/s (where 1 Å = 0.1 nm). This was followed by the formation of a 6 nm Al_2_O_3_ layer through aluminum oxidation. This layering sequence was repeated to achieve the desired HMM structure. To maximize the influence of graphene on the SPR effect, the topmost layer of the HMM consisted of Au [71].

The experimental setup for evaluating the G/HMM/D-POF sensor is shown in Figure 3b. The sensor was placed in a polyethylene (PE) reaction cell designed for probe solution detection. SPR peak shifts were monitored using a PG2000 fiber optic spectrometer, and illumination was provided by an Ocean Optics HL-2000 tungsten lamp (360–2000 nm). The surface morphology of the 3D nanostructures was analyzed using a Zeiss Gemini Ultra-55 SEM. Both numerical simulations and experimental findings indicated that the SPR peak of the sensor could be adjusted across the visible and near-infrared (NIR) spectra by altering the HMM design. The sensor demonstrated remarkable sensitivity, achieving up to 4461 nm/RIU, which makes it highly effective for bulk refractive index measurements. Moreover, it exhibited high resolution, capable of detecting DNA concentrations ranging from 10 pM to 100 nM, along with excellent linearity, repeatability, and an LOD as low as 10 pM. These characteristics underscore its significant potential for use in clinical research and medical diagnostics [71].

**Figure 4:**
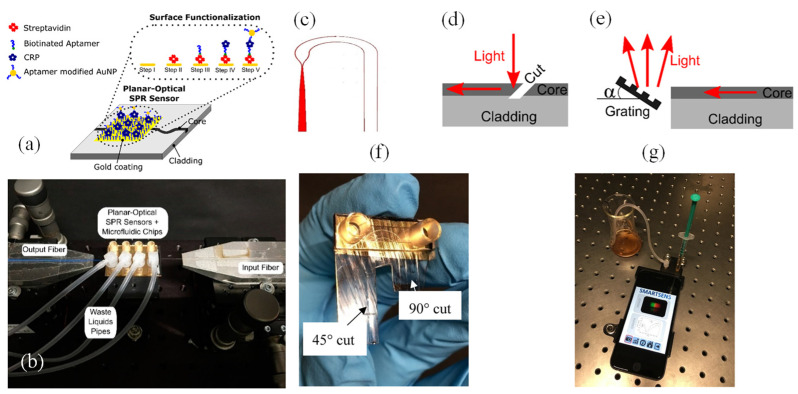
(**a**) The schematic shows the polymer-based MM planar-optical waveguide SPR sensor. A AuNP-enhanced aptamer-based sandwich assay amplifies the SPR wavelength shift caused by the binding of the target molecule, C-reactive protein (CRP) [75]. (**b**) The experimental setup includes the SPR sensor with a microfluidic chip (center) and two optical glass fibers for light coupling—one for input (right) and one for output (left) [75]. The schematic of the sensor system (**c**) illustrates the light coupling structures used for directing light into (**d**) and out of (**e**) the planar-optical waveguide sensor [79]. Light was coupled using a 45° cut and total internal reflection, while a 90° cut and a diffraction grating in reflection mode facilitated light coupling out. After assembling the coupling structures and microfluidic components (**f**), the sensor chip was placed into a 3D-printed housing (**g**) [79].

Waveguide-based SPR systems, which use planar waveguides instead of fibers, are often employed in microfluidic platforms [74,75]. These systems allow for the integration of SPR sensing with other optical techniques, such as fluorescence or interferometry, to create multifunctional devices [76,77]. This versatility makes waveguide-based SPR sensors particularly appealing for high-throughput screening and lab-on-a-chip applications [78]. Walter et al. introduced an SPR biodetection system utilizing a planar-optical multi-mode (MM) polymer waveguide structure for biomolecule detection at concentrations in the lower nanomolar (nM) range [75]. The configuration of the proposed sensor is illustrated in Figure 4a. The SPR sensor spectrum was recorded in transmission mode using an affordable white light LED (Thorlabs MCWHF2) and an optical spectrometer (Avantes AvaSpec-3648, Avantes, Apeldoorn, The Netherlands). Optical glass fibers were employed to couple light into and out of the planar-optical SPR waveguide sensor. For input coupling, tapered graded-index multimode (MM) optical glass fibers (OM4) with a 25 µm spot diameter (Thorlabs LFM100, Thorlabs, Newton, NJ, USA) were utilized. A step-index MM fiber with a numerical aperture of NA = 0.5 (Thorlabs FP200URT, Thorlabs, Newton, NJ, USA) was used to collect light exiting the waveguide structure. Linear stages (Thorlabs RBL13D/M, Thorlabs, Newton, NJ, USA) facilitated the precise alignment of the optical fibers concerning the planar-optical SPR waveguide sensor. Figure 4b depicts the experimental setup, including the planar-optical waveguide SPR sensor integrated with a microfluidic chip and the arrangement of optical fibers for light coupling.

The sensor demonstrated a sensitivity of 608.6 nm/RIU to RI variations, with a measurement resolution of 4.3 × 10^−3^ RIU. By integrating the SPR sensor with an aptamer-functionalized, AuNP-enhanced sandwich assay, it successfully detected C-reactive protein (CRP) in buffer solutions, yielding a response of 0.118 nm/nM. The MM polymer waveguide’s design and straightforward implementation make this biodetection system an excellent candidate for cost-effective, disposable lab-on-a-chip systems. It was particularly suited for rapid, multiplexed biomarker detection on a single integrated platform using simple and economical devices [75].

Walter et al. presented a groundbreaking all-optical plasmonic sensor platform designed for seamless smartphone integration. This system utilized planar-optical waveguide structures embedded within a polymer chip [79]. Its biosensing capability was demonstrated by detecting 25-hydroxyvitamin D (25OHD) in human serum samples via a AuNP-enhanced aptamer-based assay. The schematic of the planar-optical waveguide structure employed in the design is depicted in Figure 4c. To facilitate light transfer, coupling structures (Figure 4d,e) enabled the smartphone’s flashlight LED to inject light into the planar-optical sensor and guide it back to the smartphone camera. A 45° incision, made with a razor blade, was crucial for coupling light into the system. This cut created an air gap, causing total internal reflection at the polymer/air boundary and channeling light into the waveguide. The 45° orientation ensured efficient perpendicular coupling between the flashlight LED and the planar-optical sensor.

To direct light into the smartphone camera, waveguides were precisely cut perpendicular to their length using a razor blade heated to 65 °C. The guided light was subsequently reflected onto a diffraction grating, operating in reflection mode, which dispersed the light into the smartphone camera for spectral analysis. This grating was created by replicating Thorlabs diffraction grating (GH13-18V, 1800 lines/mm, Thorlabs, Newton, NJ, USA) and was coated with a 100 nm thin Ag film using a sputtering process to enhance its reflective properties. The grating’s angle (α) was set at 7°, as shown in Figure 4e, and its precise positioning within the sensor housing was achieved through 3D printing. This configuration ensured that the smartphone camera could capture the full spectrum emitted by the flashlight LED, despite the limitations of its aperture.

For fluid handling, syringes (B.Braun Injekt-F 1 mL) and silicone tubing (Ibidi elbow male Luer adapter with silicone tubing) were utilized. These components were connected to Luer adapters on the sensor’s microfluidic chip to introduce liquids for surface modifications on the Au layer of the SPR sensor, and to remove waste liquids from the system. Figure 4f,g display the assembled sensor chip and the complete experimental setup. The sensor achieved a sensitivity of 0.752 pixels/nM for detecting 25OHD concentrations in the range of 0–100 nM.

The waveguide design supported system miniaturization and parallelization, allowing for the simultaneous detection of multiple biomarkers. All optical elements were integrated into a single polymer chip, making the design conducive to large-scale, cost-efficient production. By leveraging the ubiquity of smartphones, this approach holds immense promise for lab-on-chip applications [79].

#### 2.2.3. Nanoarray-Based and Localized SPR (LSPR)

Nanoarray-based SPR and localized SPR (LSPR) are advanced configurations that leverage nanostructures to enhance the sensitivity and specificity of plasmonic sensing [80,81]. In these configurations, metallic nanoparticles or nanostructured arrays are used to confine SPs to nanoscale regions. Unlike traditional SPR, where SPs propagate along a continuous metal–dielectric interface, LSPR involves localized oscillations of conduction electrons within individual nanoparticles. LSPR offers several unique advantages, including sensitivity to changes near the nanostructures [82]. This property enables the detection of extremely small quantities of analytes, as even minor changes in the RI near the nanoparticles cause significant shifts in the resonance peak. Additionally, LSPR systems are inherently compact and can be integrated into micro- and nano-scale devices [83]. However, LSPR sensors often exhibit low figures of merit (FOM), typically below 5 RIU^−1^, due to the inherent radiation losses caused by the random arrangement and localization of nanoparticles. These losses can be mitigated by manipulating the phase of the scattered field through adjustments to the structural parameters of nanoparticle arrays.

Wang et al. proposed a 2D periodic crescent nanoarray-based surface lattice resonance (SLR) sensor designed to achieve a high FOM [84]. Through mode field analysis and the optimization of structural parameters, key findings were obtained. The SLR spectrum was found to exhibit two distinct line shapes: a Fano-like line with an FOM on the order of 101 and a separate line reaching an FOM of 103. Additionally, the relative size of the excitation wavelengths between SLR and LSPR was identified as critical; while a higher relative size increases the FOM, it also leads to a more rapid decrease in resonance depth. The suggested crescent nanoarray achieved an FWHM of less than 0.5 nm and an FOM exceeding 1000 RIU^−1^, with a Q-factor surpassing 3000. These results demonstrate the significant potential of plasmonic nanoarray-based SLR structures for ultra-sensitive trace substance detection [84].

Nanoarray-based SPR sensors, which use ordered arrays of nanostructures, provide additional benefits, such as enhanced signal uniformity and reproducibility. These arrays can be fabricated using techniques like EBL or NIL, enabling precise control over the size, shape, and spacing of the nanostructures [85]. By optimizing these parameters, researchers can tune the plasmonic response to achieve maximum sensitivity for specific applications. SPR and LSPR effects have served as foundational principles for developing highly sensitive sensors in recent decades. Advances in nanofabrication technology have enabled the widespread use of plasmonic nanoarray sensors based on these phenomena in chemical and biological analyses. By leveraging surface-enhanced fields and detecting RI changes, these sensors can quantitatively and qualitatively identify analytes. Recent developments in ultrasensitive plasmonic biodetection systems have led to the creation of high-performance platforms for diverse biomedical applications, including point-of-care diagnostics and personalized medicine. Additionally, integrating plasmonic nanoarrays with electrochemical sensing has expanded their application scope and enhanced sensing precision [85].

A rapid, portable, and cost-effective method for detecting SARS-CoV-2 infection is crucial for managing the COVID-19 pandemic. Yang et al. proposed an LSPR sensor based on a silver nanotriangle (AgNT) array functionalized with human angiotensin-converting enzyme 2 (ACE2) protein, which was developed for efficient coronavirus detection [86]. The sensor demonstrated high sensitivity and specificity when tested with the SARS-CoV-2 spike RBD protein and CoV NL63 virus. A linear relationship was observed between the LSPR wavelength shift and the logarithmic concentration of spike RBD protein and CoV NL63. The LODs for spike RBD protein, CoV NL63 in buffer, and untreated saliva were 0.83 pM, 391 PFU/mL, and 625 PFU/mL, respectively, with a detection time of under 20 min. These findings suggested that the AgNT array optical sensor has strong potential use as a rapid point-of-care diagnostic tool for COVID-19 [86].

**Figure 5:**
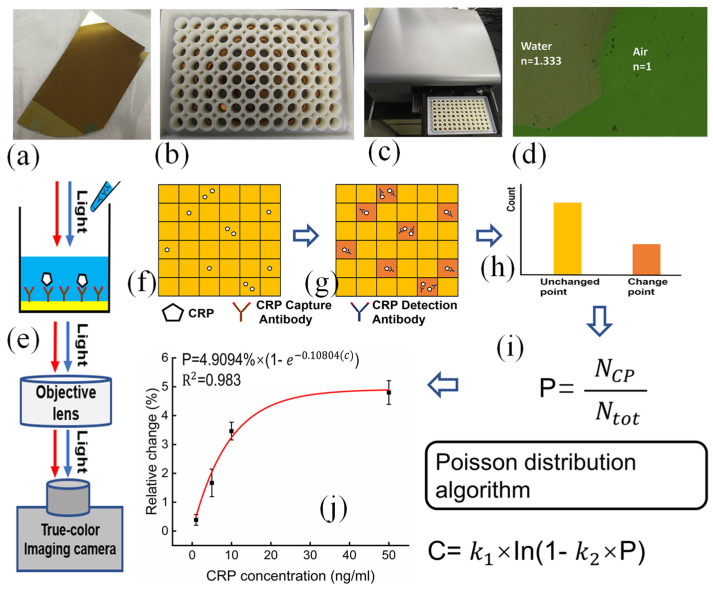
Overview of the Au–TiO_2_–Au nanocup array chip and its use: (**a**) A photograph of a single Au–TiO_2_–Au nanocup array chip. (**b**) Integration of the chip into a custom-made 96-well plate. (**c**) Testing using a standard microplate reader with small sample volumes. (**d**) Transmission microscopy image showing different colors, green for air and olive for water, on the chip surface [87]. (**e**–**j**) The mechanism of the digital plasmonic immunosorbent assay for protein binding kinetics [88]; (**e**) schematic of the optical detection system setup, (**f**) random distribution of diluted CRP proteins on the device surface via binding to CRP capture antibodies, (**g**) binding of detecting antibodies to captured CRP causes a red shift in the peak resonance wavelength in transmission intensity, (**h**) Ppixel comparison between CRP and blank solution binding areas using image analysis, (**i**) digital SPR calculations are based on the Poisson distribution used in digital PCR, (**j**) plot showing relative count changes as a function of CRP concentration, calculated using Digital SPR arithmetic [88].

Conventional colorimetric and fluorescence assays used with generic microplate readers are generally incapable of performing dynamic measurements of protein–protein interplays or quantifying the kinetic association and dissociation constants of these interplays. In contrast, such kinetic analyses are typically restricted to specialized and costly SPR equipment. Dang et al. introduced an innovative approach by integrating coupled plasmonic–photonic resonance nanosensors into a standard 96-well plate format (Figure 5a–c) [87]. Figure 5d illustrates that the sensor chip displays varying colors depending on the medium applied. For the first time, this enabled label-free, SPR-like dynamic protein binding measurements and kinetic quantification using a conventional microplate reader. The cost-effective nanosensor plate achieved the highly sensitive detection of immobilized protein interplays through changes in transmission optical density (OD) at specific wavelengths, which are recorded via a microplate reader. The relative end-point OD changes exhibit a strong linear correlation with protein concentrations ranging from 0.05 to 50 μg/mL, and protein quantification results in serum aligning closely with those from hospital laboratory tests. Crucially, the kinetic association and dissociation constants of protein interplays can be determined through time-lapse OD measurements in the generic microplate reader. This development makes SPR-like protein binding kinetics analysis accessible to a broad range of chemistry and biomedical laboratories equipped with standard microplate readers [87].

A Au–titanium plasmonic nanopore array-based imaging sensor and digital plasma immunoassay enable highly sensitive protein detection. By combining Poisson statistical algorithms with digital SPR imaging, the device detects low concentrations of C-reactive protein (CRP) and its antibody binding, achieving an LOD of 2.36 ng/mL using white light [88]. It also determines equilibrium dissociation constants through the dynamic imaging of CRP-antibody interplays. This approach eliminates complex spectroscopy, offering a promising portable optical sensing method for early disease detection using visible light. Figure 5e presents a schematic of the detection apparatus. The use of the Poisson distribution algorithm was introduced from digital PCR technology integrated with SPR imaging for the first time (Figure 5f–i), enabling the quantification of CRP concentrations via digital SPR imaging (Figure 5j) [88].

## 3. Key Components of SPR-Based Biodetection Systems

SPR biodetection systems are powerful analytical tools used in biological, chemical, and medical research for the real-time, label-free detection of biomolecular interactions. The performance and reliability of SPR biosensors depend heavily on three primary components: metallic layers, surface chemistry and functionalization, and optical setup and instrumentation. Below, each component is explored in detail.

### 3.1. Metallic Layers

The metallic layers are fundamental to the phenomenon of SPR and warrant detailed examination, as their characteristics directly impact the occurrence of SPR and their utility in anchoring biomolecules [89]. These layers often consist of noble metals like Au [90,91] or Ag [92] due to their high electron density and plasmonic properties. It is crucial to specify whether the metals should be in nanoparticle form or present as continuous films, as this choice significantly influences the sensitivity and efficiency of SPR. For instance, nanoparticles provide enhanced surface area and tunable plasmonic properties, but their shape, size, and spatial arrangement must be carefully controlled [93]. Spherical nanoparticles, nanorods, or even more complex geometries can exhibit distinct SPR signatures, and variations in size can shift resonance frequencies, thus affecting the system’s response. Additionally, the arrangement of nanoparticles—random versus ordered patterns—plays a role in plasmon coupling, which can amplify or dampen SPR signals [94]. These factors also critically determine the anchoring and functionalization of biomolecules, as the surface morphology and chemical properties of the metallic layers influence how biomolecules attach and remain stable. Addressing these details is essential to optimizing both SPR performance and the effectiveness of biomolecular interactions in applications like biosensing and diagnostics [95].

In recent years, alternative materials like Al, Cu, and multilayer composites have been investigated to improve SPR sensor performance [96,97]. These materials are often combined with protective coatings to balance sensitivity and durability. For example, a Au–Al bilayer structure can optimize resonance sharpness while enhancing longevity. The thickness of the metallic layer, typically in the range of 40–60 nanometers, is also critical, as it directly influences the resonance conditions. The optimization of material properties and thickness ensures enhanced SPR signals and better sensitivity, enabling the accurate detection of low-abundance analytes. The resolution of SPR sensors in Kretschmann geometry was numerically simulated for varying thicknesses of metallic layers composed of Ag, Cu, and Al, combined with a Au coating layer, across a range of wavelengths. The analysis considered the detection of RI changes in the bulk medium and variations in the optical thickness of an adsorption layer. Among the evaluated sensor configurations, the lowest resolution was observed with a single Al layer in the ultraviolet region and a single Au layer at longer wavelengths [98].

Radha et al. investigated multiple multilayer metallic SPR biosensor designs incorporating Au, Ag, Al, and Cu, using an N-layer matrix formalism tailored to fixed-angle spectral SPR sensing [99]. Sensor configurations were evaluated based on stringent criteria for sensitivity, detection accuracy, and FOM. The results reveal that three- and four-layer configurations utilizing Al and Cu achieve the highest FOM among configurations meeting the established benchmarks. Notably, the four-layer Al/Cu/Al/Cu sensor exhibited the maximum FOM of 1433.82/RIU for an analyte RI of 1.408. These sensors were particularly effective for analytes with refractive indices within the range of 1.350–1.414.

### 3.2. Surface Chemistry and Functionalization

The surface chemistry of the metallic layer plays a pivotal role in ensuring specific and stable biomolecule immobilization [100]. Functionalization techniques enhance the selectivity of SPR biodetection systems, allowing them to differentiate target analytes from complex sample matrices. One widely employed strategy is the use of thiol-based SAMs [101]. Thiols form strong covalent bonds with Au, creating a well-ordered, functionalized layer that can anchor various biomolecules like antibodies, DNA, or aptamers [102].

Covalent linkages are another approach for robust immobilization. Techniques involving carbodiimide chemistry (e.g., EDC/NHS coupling) or click chemistry enable the precise attachment of biomolecules to the sensor surface. To further enhance specificity and reduce non-specific binding, anti-fouling coatings are employed. Polyethylene glycol (PEG) layers, zwitterionic polymers, and hydrophilic SAMs are examples of coatings that resist the adsorption of unwanted proteins and other contaminants, thereby improving the biosensor’s overall performance. These surface modifications ensure high reproducibility, stability, and sensitivity in detection applications.

Accurately determining and maintaining resonance conditions in SPR-based biosensors in complex biological environments requires careful consideration of both physical and chemical factors [103]. In such environments, the presence of multiple biomolecules, ions, and other interfering substances can alter the refractive index near the sensor surface, complicating the detection of specific binding events [104]. To address this, it is essential to calibrate the SPR system using known standards and buffer solutions that mimic the biological environment while minimizing non-specific interactions [105]. Regular baseline monitoring helps account for gradual shifts in resonance due to environmental changes. Functionalizing the sensor surface with highly specific ligands or antibodies reduces cross-reactivity and ensures selectivity [106]. Additionally, advanced data processing techniques, such as real-time referencing and multivariate analysis, can differentiate specific binding signals from background noise. Maintaining precise temperature control and ionic strength in the sample can further stabilize resonance conditions, ensuring consistent and reliable sensor performance [107]. For instance, Vala et al. developed a compact SPR sensor capable of detecting up to 10 analytes simultaneously [108]. It uses angular interrogation on a Au-coated diffraction grating within a cartridge with 10 fluidic channels. Optimized for resolution and compactness, the sensor achieved 6 × 10^−7^ RIU in refractometric tests and detected oligonucleotides at concentrations below 1 nM, showcasing its multiplexed detection capability.

Moreover, mode-multiplexed miniaturized sensors enable the real-time detection of multiple biomolecules. Haider et al. introduced a grapefruit PCF-based mode-multiplex SPR sensor for simultaneous multi-analyte detection [109]. The sensor used three grapefruit-shaped air holes coated with a Au layer as independent detection channels. Finite element method (FEM) analysis and stack-and-draw fabrication optimized its design. For the x-polarized mode, channels one and three achieved a sensitivity of 2000 nm/RIU and 18,000 nm/RIU at refractive indices of 1.34 and 1.41, while channel two reached 3000 nm/RIU at 1.36.

Islam et al. explored the potential use of novel hexameric peptide ligands for on-line IgG detection in bioprocesses [110]. SPR was employed to investigate the binding interplays between human IgG and the hexameric peptide ligand HWRGWV, which was covalently attached to alkanethiol SAMs on Au surfaces. The peptide’s coupling to SAMs was confirmed, and peptides with either removable Fmoc or acetylated N-termini were covalently grafted via their C-termini to create active peptide SPR sensors. These sensors were subsequently tested for their IgG binding properties. The binding dynamics and extent of peptide–IgG interactions were compared to a conventional system featuring protein A immobilized on a Au surface through disulfide monolayers. The protein A-based system exhibited an equilibrium dissociation constant of 1.4 × 10^−7^ M. In contrast, the acetylated peptide version (Ac-HWRGWV) immobilized on alkanethiol SAMs demonstrated a dissociation constant of 5.8 × 10^−7^ M, while HWRGWV on alkanethiol SAMs (following Fmoc-HWRGWVA deprotection) showed a dissociation constant of 1.2 × 10^−6^ M. Maximum IgG binding capacities (Q_m_) were determined as 6.7, 3.8, and 4.1 mg m^−2^ for protein A and the two HWRGWV-based biodetection systems, respectively.

The kinetic analysis of real-time adsorption data provided insights into the apparent rate constants for adsorption and desorption. The findings reveal that peptide–IgG binding followed a reaction-controlled mechanism, whereas protein A–IgG binding exhibited partial mass transfer (diffusion) control. Furthermore, the adsorption rate constant (k_a_) for protein A varied inversely with IgG concentration, while the peptide ligand displayed consistent ka values across different IgG concentrations and flow rates. These observations shed light on the distinct mechanisms governing IgG binding to protein and peptide ligands [110].

### 3.3. Optical Setup and Instrumentation

The optical setup is the backbone of SPR-based biodetection systems, determining their functionality and versatility. Traditional SPR systems employ a Kretschmann configuration, where a prism couples light into the metallic layer. Modern SPR imaging (SPRi) systems extend this principle, enabling the spatially resolved detection of multiple analytes on a single sensor surface. SPRi combines plasmonic resonance with imaging techniques, allowing the simultaneous monitoring of multiple interplays, making it ideal for high-throughput screening. Advances in miniaturized and portable SPR devices have made SPR technology more accessible for point-of-care diagnostics and field applications [111,112,113]. These systems leverage microfluidics, compact light sources (e.g., LEDs or VCSELs), and sensitive detectors to achieve portability without compromising sensitivity. Integration with smartphone-based readouts and wireless data transmission has further expanded the usability of SPR biosensors, facilitating their deployment in resource-limited settings. Emerging trends in chip-based SPR sensors and nanophotonic designs are further pushing the boundaries of miniaturization and affordability [114,115].

**Figure 6:**
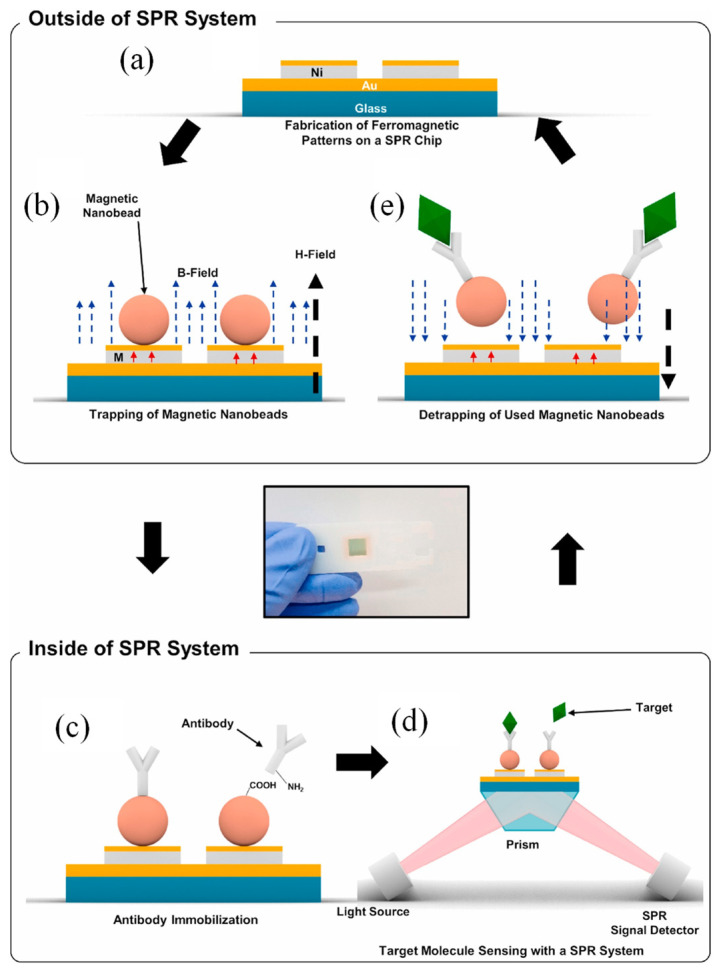
Illustration of the cyclic process for repeated sensing measurements using the reusable SPR biodetection system chip: (**a**) The SPR chip incorporates ferromagnetic Ni patterns integrated with a standard SPR chip design [116]. (**b**) Magnetic particles are captured on the SPR chip under the influence of an external magnetic field [116]. (**c**) Antibodies are immobilized on the magnetic particles via EDC-NHS coupling in the SPR system [116]. (**d**) Target molecules are detected [116]. (**e**) Magnetic particles are released by reversing the external magnetic field [116].

For instance, Yoo et al. developed a reusable magnetic SPR sensor chip designed for the repeated detection of various target molecules using a conventional SPR system [116]. The reusable SPR sensor chip enabled repeated sensing through a straightforward process (Figure 6). The fabrication involved depositing a metal film (45 nm Au on 5 nm Cr) onto a glass substrate, followed by the creation of Ni/Au pattern arrays (10 nm Au on 50 nm Ni, 5 μm × 10 μm) (Figure 6a). The Au layer prevented Ni oxidation and facilitated biochemical molecule binding for SPR sensing. The substrate was mounted onto an SPR measurement system (Biacore, GE Healthcare) using double-sided adhesive tape. Magnetic particles functionalized with carboxylic groups were introduced onto the chip, and a perpendicular magnetic field (150 mT) magnetized the Ni patterns, attracting and trapping the particles (Figure 6b). Excess particles were removed via PBS washing, leaving only strongly bound particles. These remained attached even without an external magnetic field. Antibodies were immobilized onto trapped particles using EDC and NHS, preparing the chip for specific target molecule detection (Figure 6c). Changes in the RI near the chip surface were measured in resonance units (Figure 6d). Post-sensing, an external magnetic field in the opposite direction weakened the magnetic field over the Ni patterns, allowing trapped particles to be removed with DI water (Figure 6e). This refreshed the chip surface without chemical treatments, reducing contamination. The process eliminated the need for continuous magnetic fields during sensing, making the reusable chip compatible with conventional SPR systems [116].

Together, the careful design of metallic layers, surface functionalization, and optical instrumentation ensures that SPR-based biodetection systems continue to provide highly sensitive and specific detection capabilities for a wide range of applications, from clinical diagnostics to environmental monitoring.

### 3.4. Impact of Laser Wavelength on SPR Detection

The wavelength of the laser used in an SPR system plays a pivotal role in determining the detection sensitivity and accuracy, as it directly affects the excitation of SPs at the metal–dielectric interface [117]. SPs are collective oscillations of free electrons at the surface of a thin metal film, and their excitation depends on the resonance condition between the incident light and the surface plasmons. This condition is highly wavelength-dependent, as different wavelengths interact with the optical properties of the metal film and the analyte medium in distinct ways. Au, the most commonly used metal for SPR applications, exhibits strong plasmonic behavior in the visible and NIR regions [118,119]. Consequently, lasers with wavelengths typically ranging from 600 nm to 900 nm are most effective for exciting SPs. Shorter wavelengths, such as those in the UV range, are less commonly used due to increased absorption and scattering losses in the metal film, which can reduce detection sensitivity. On the other hand, longer wavelengths, particularly in the NIR range, may be beneficial for detecting larger biomolecules or for applications involving samples with higher refractive indices, as they provide a deeper penetration of the evanescent field.

The choice between a fixed-wavelength and a tunable-wavelength laser depends on the specific requirements of the SPR application [120,121]. Fixed-wavelength lasers are simpler to integrate, more cost-effective, and suitable for standard applications where the refractive index changes of the sample are well understood. They are often employed when the system is designed for a particular type of analyte or a narrow range of detection conditions. However, tunable-wavelength lasers offer greater flexibility and precision, as they allow users to adjust the wavelength to achieve optimal resonance for varying sample types or experimental conditions [113]. This is particularly advantageous for multiplexed detection or when analyzing substances with diverse optical properties. The intensity of the laser used in an SPR system is another critical parameter, as it influences the signal-to-noise ratio (SNR) and overall sensitivity. While SPR systems do not impose stringent intensity requirements, the laser should provide sufficient power to generate a clear and stable plasmonic resonance signal without introducing excess noise or damaging the sample. In most cases, laser intensities ranging from a few milliwatts to tens of milliwatts are adequate. However, the specific intensity required depends on factors such as the optical setup, the quality of the metal film, and the sensitivity of the detection system. Low-intensity lasers may be preferable for delicate samples to avoid photodamage, while higher intensities might be necessary for robust systems or when working with highly absorbing media.

## 4. Applications for SPR-Based Biodetection Systems

SPR-based biosensors have emerged as a versatile and sensitive platform for real-time, label-free detection in various fields. Their ability to monitor biomolecular interactions with high specificity and precision has led to their adoption in diverse applications, ranging from healthcare to environmental safety and beyond. Table 1 captures the versatility of SPR-based sensors across a wide range of fields, emphasizing their adaptability and efficiency in addressing diverse analytical challenges.

### 4.1. Biomedical Diagnostics

SPR-based biodetection systems are widely utilized in biomedical diagnostics due to their ability to detect biomarkers, DNA/RNA sequences, and pathogens with exceptional sensitivity [122]. These biosensors enable the early detection of diseases by identifying specific biomarkers such as proteins or nucleic acids associated with conditions like cancer, cardiovascular diseases, and infectious diseases. They play a pivotal role in personalized medicine, tailoring treatments to individual patients by analyzing biomarkers that indicate drug responsiveness [123]. Additionally, their compact designs and ease of use make them suitable for point-of-care diagnostics, offering rapid and reliable results in clinical and field settings. This portability significantly improves healthcare delivery in remote or resource-limited areas [67,111].

The development and deployment of sensors for detecting biomolecules in clinical samples represents a central objective within the sensing research field. Techniques like SPR, along with related methods such as LSPR and imaging SPR, have progressed significantly, achieving a level of sophistication that enables their use in biomolecule detection in clinical settings. Recent advancements have demonstrated the utility of SPR-based approaches for analyzing antibodies, proteins, enzymes, drugs, small molecules, peptides, and nucleic acids in biological fluids from patients with various medical conditions. These include Alzheimer’s disease, hepatitis, diabetes, leukemia, and cancers such as prostate and breast cancer, among others [124].

**Figure 7:**
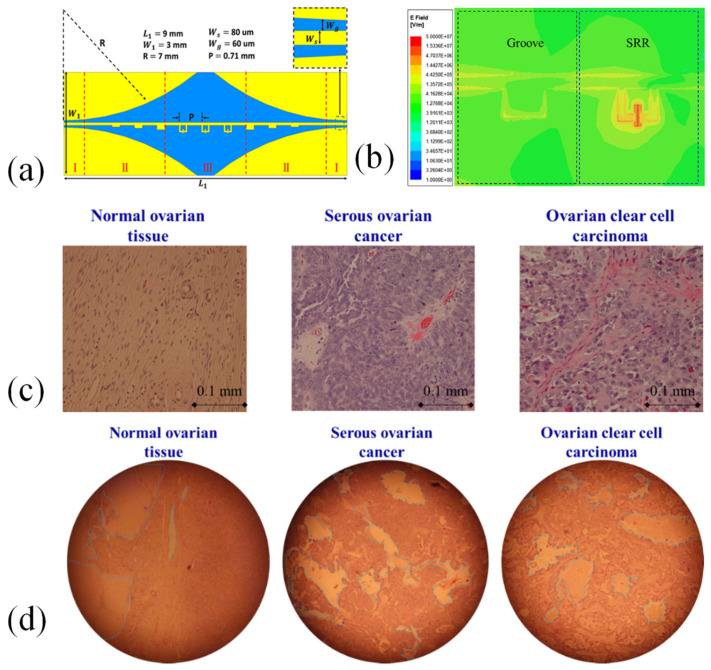
(**a**) Spoof SPP biodetection system featuring in-series SRRs. (**b**) Electric field distribution for the groove and SRR in the proposed design at 53.99 GHz. (**c**) Microscope images of stained tissues: normal, serous ovarian cancer, and ovarian clear cell carcinoma. (**d**) Zoomed-out views of these tissues [125].

Artificial periodic grooves on metal surfaces can simulate optical SPPs with a high cutoff frequency in the microwave range, often referred to as spoof SPPs. This phenomenon has gained considerable interest in biosensing due to its ability to significantly enhance localized electric fields. Zhang et al. developed an innovative SPP-based biodetection system using split-ring resonators (SRRs) for detecting ovarian cancer (Figure 7a) [125]. The SRRs are placed in series on a metal line, replacing the traditional periodic grooves, while maintaining the same cutoff frequency and generating a sharp resonance. This resonance amplifies the localized electric field by a factor of 250, greatly improving the sensor’s sensitivity to changes in permittivity. The electric field distribution for both the groove and SRR designs at 53.99 GHz is illustrated in Figure 7b [125].

Figure 7c shows the stained cells of normal, SOC, and OCCC tissues, highlighting distinct differences in cell size and uniformity. Notably, the cancer cells are larger compared to the normal cells. Among them, the OCCC cells exhibit the largest size and the most irregular distribution. In Figure 7d, the zoomed-out images of these three tissue types are presented, with each image having a diameter of 3.25 mm and an area of 8.2 mm^2^. The sensor’s sensitivity, in terms of frequency shift, was initially evaluated through electromagnetic simulations and subsequently tested using sucrose solutions at various concentrations. Thanks to its high sensitivity, the biodetection system was successfully employed to detect serous ovarian cancer (SOC) and ovarian clear cell carcinoma (OCCC) tissues. The intrinsic resonant frequency of the biosensor was 53.990 GHz, shifting to 53.814–53.968 GHz for normal tissues, 53.698–53.872 GHz for SOC, and 53.719–53.845 GHz for OCCC. The average resonant frequency of all ovarian cancer tissues was 53.812 GHz, slightly lower than that of normal tissues, offering a useful reference for cancer detection. Additionally, the average transmittance of cancerous tissues was lower than that of SOC tissues, which could assist in differentiating between SOC and OCCC. The proposed planar SPP biodetection system provides a rapid, highly sensitive, and label-free method for ovarian cancer detection [125].

### 4.2. Pharmaceutical and Drug Discovery

The pharmaceutical industry leverages SPR biodetection systems for the kinetic analysis of molecular interactions, including drug–target binding, antibody–antigen recognition, and receptor–ligand dynamics [126,127,128]. These interactions are crucial in understanding drug efficacy and safety. SPR enables the high-throughput screening of drug candidates by rapidly evaluating binding affinities and mechanisms of action, thus accelerating the drug development pipeline [129]. The non-invasive, real-time capabilities of SPR provide insights into the thermodynamic parameters of interactions, guiding the optimization of lead compounds and enhancing the efficiency of drug discovery processes [130].

SPR is an advanced analytical method widely employed to study the interactions between biomolecules, including proteins and nucleic acids. This technique has proven invaluable in assessing the binding affinity of novel therapeutics, such as small molecules and biomolecule-derived drugs, for a range of conditions, including lupus, thrombin inhibition, HIV protease inhibition, and DNA gyrase inhibition, among others [127,131]. Recently, there has been growing interest in nanotherapeutics (nanoRx), owing to their unique capabilities for controlled drug release and targeted delivery to diseased tissues [132,133,134]. NanoRx has significant promise for addressing various drug delivery challenges, as they enable precise molecular interplays between surface molecules on the nanoparticles and those in the targeted tissues, reducing off-target effects on healthy tissues [135]. The success of nanoRx largely depends on carefully managing their interplays and binding properties within the body. Given the potential of nanoRx to facilitate specific molecular engagements, SPR is an effective tool for rapidly evaluating small quantities of nanoRx formulations to assess both desired and unintended molecular interactions. In the future, incorporating SPR into the design and screening processes for nanoRx could significantly enhance the development of targeted formulations and improve their therapeutic effectiveness [131,132,136].

Nanomedicines, such as polymer nanocarriers with controlled drug release mechanisms, represent the next generation of therapeutic agents, offering enhanced treatment effectiveness and minimized side effects. To ensure their safety and efficiency, accurately assessing drug release kinetics is essential. Libanska et al. explored the use of various analytical techniques, including SPR biodetection system technology, capillary electrophoresis, and ^1^H diffusion-ordered nuclear magnetic resonance spectroscopy, which were applied for drug release analysis [137]. These methods were optimized to measure the pH-triggered release of three drugs with different molecular structures from a polymeric carrier. The suitability of these techniques was evaluated and compared for characterizing drug release, considering factors such as their applicability to different sample types, the biological relevance of the experimental conditions, method complexity, and the quality of the data obtained. Among the methods tested, SPR emerged as the most versatile, enabling the continuous monitoring of drug release in a flow-through system and requiring only a small sample volume [137].

Khokhlova et al. suggested that SPR might explain the strong oxidative effects of low-intensity laser irradiation in living systems [138]. A 1265 nm laser was shown to induce significant ROS production in HCT116 and CHO-K1 cell cultures, attributed to localized plasmon-polaritons on mitochondrial cristae. Low-intensity irradiation, a narrow laser bandwidth (<4 nm), and small biological structures (~10 μm) enabled plasmon-polariton formation and strong laser field confinement, leading to oxidative stress [138].

The high-risk HPV E7 protein promotes the degradation of the retinoblastoma tumor suppressor protein (RB) through direct interaction, making it a target for anticancer drug development. To enable the high-throughput screening of RB-E7 interaction inhibitors, an SPR imaging-based protein array chip was developed [139]. The chip featured GST-E7 immobilized on an Au surface functionalized for GST-tagged proteins. His6-RB was applied via a microarrayer, and its interactions were monitored using SPR imaging. Increasing His6-RB concentrations in the spotting solution resulted in proportional SPR signal increases, confirming concentration-dependent binding [139].

To evaluate the inhibition of this interaction, His6-RB solutions containing a peptide (PepC) derived from an E7 motif were tested. SPR imaging results demonstrate that PepC effectively inhibited the GST-E7/His6-RB interaction in a dose-dependent manner. These findings highlight the utility of SPR imaging-based protein array chips for screening small molecule inhibitors of protein–protein interactions [139].

### 4.3. Environmental Monitoring

In the realm of environmental monitoring, SPR-based biosensors serve as powerful tools for detecting pollutants, toxins, and other hazardous substances [3]. They can identify heavy metals, pesticides, and endocrine-disrupting chemicals in water, soil, and air, ensuring compliance with environmental regulations [140]. Furthermore, SPR biodetection systems are instrumental in water quality monitoring, where they detect microbial contamination and chemical pollutants with high accuracy. Their rapid response times and ability to perform continuous monitoring make them invaluable for maintaining ecological balance and protecting public health [141].

A Kretschmann-configured SPR gas sensor was proposed to enhance sensitivity, incorporating a monolayer of MXene (Ti_3_C_2_Tx), a bimetallic layer of Ag and Au, and 40 layers of black phosphorus (BP) [142]. A sodium fluoride (NaF) glass prism was used as the coupling substrate. The sensor’s performance was evaluated through the angular interrogation technique. The BP surface served as an ideal platform for gas molecule attachment, leveraging its distinct binding capabilities. The proposed sensor achieved a peak sensitivity of 248°/RIU at an R_min_ value of 6.5 × 10^−4^ (a.u.) and demonstrated an outstanding figure of merit. Numerical simulations revealed that incorporating multilayer BP significantly enhanced the sensor’s performance compared to conventional designs. Furthermore, the sensor has been successfully demonstrated in detecting a range of gases, from helium to carbon dioxide [142].

Yang et al. investigated the significant enhancement of the sub-wavelength transverse displacement in the photonic spin Hall effect (PSHE) using surface exciton polaritons (SEPs), with a focus on applications in gas sensing [143]. By leveraging SEPs, a transverse displacement equivalent to 14.4 times the incident light wavelength was achieved, which was nearly three times larger than that obtained with SPR-enhanced PSHE. A gas sensor utilizing SEP-enhanced PSHE was introduced for detecting SO_2_, achieving an RI sensitivity of 6320.4 µm/RIU within an RI range of 1.00027281 to 1.00095981. These findings highlight the potential of SEPs as an effective mechanism for enhancing PSHE and paving the way for advancements in highly sensitive gas, biosensing, and chemical sensing technologies [143].

Proenca et al. introduced a novel parameter known as LSPR gas sensitivity to evaluate the performance of plasmonic gas sensors [144]. This model incorporated both the surface sensitivity and the plasmon decay length, linking the LSPR response during gas exchange to an equivalent RI change corresponding to the adsorbed gas layers. To demonstrate the application of this parameter, ellipsoid-shaped AuNPs were arranged in tightly packed hexagonal lattices. These sensors provided numerous benefits, including finely tunable interparticle gaps (ranging from 18–29 nm) between nanoparticles with diameters of 72–88 nm, and a reliable, scalable fabrication method that maintained a consistent arrangement over large surface areas (up to several cm^2^). The LSPR response was assessed by using a sensor system that cycled between various inorganic gases, such as He/Ar and Ar/CO_2_, at constant pressure and room temperature. The findings confirmed that this parameter is effective for benchmarking plasmonic gas sensors, demonstrating their independence from gas type or pressure, regardless of the sensor’s structure. Furthermore, it helped resolve inconsistencies often encountered when comparing the performances of plasmonic sensors in liquid and gas phases [144].

**Figure 8:**
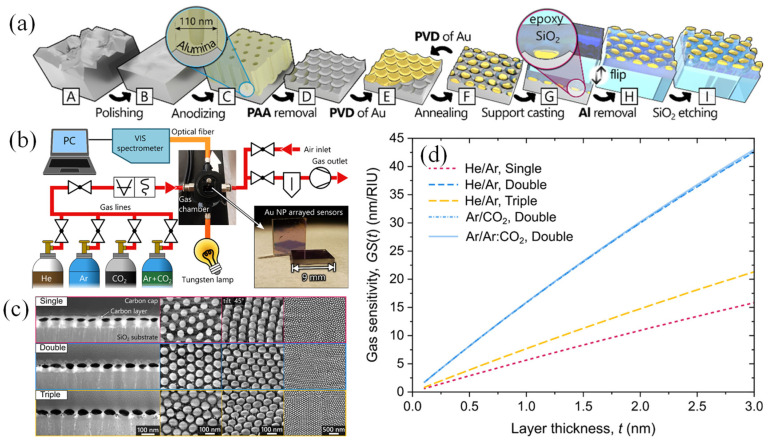
(**a**) A schematic of the AuNP layer fabrication process (**A**–**I**) [144]. (**b**) A schematic of the HR-LSPR spectroscopy system is shown, which includes a gas chamber connected to gas controls, a vacuum unit, and gas cylinders. The gas chamber has optical and gas in/out ports, along with a holder for the plasmonic sensor [144]. (**c**) STEM (cross-sectional) and SEM images of three different Au NP arrangements on SiO_2_ nanopillars [144]. (**d**) LSPR gas sensitivity functions GS(t) for the three nanoparticle arrangements, calculated for various gas exchanges [144].

The fabrication steps of the sensor are outlined in Figure 8a, with the individual procedures from A to I described in detail. AuNP layers were deposited onto Al templates with a ~110 nm cell size and three distinct size distributions. A 250 μm-thick aluminum sheet (99.999% purity) was cut into 25 × 50 mm^2^ sections, then cleaned, annealed at 550 °C in a vacuum (~10^−4^ Pa, 15 h), and polished mechanically and electrochemically. The aluminum sheets were anodized at 40 V in a 0.3 M oxalic acid solution at 8 °C for 20 h to produce a porous anodic alumina (PAA) surface, which was later removed to create a nanobowled aluminum template.

AuNP layers were fabricated using the solid-state dewetting of a magnetron-sputtered gold film. Single (8 nm), double (8 + 6.5 nm), and triple (8 + 6.5 + 5 nm) size distributions were achieved through repeated thin-film deposition (0.35 × 10^−1^ nm/s, 10^−1^ Pa) followed by annealing at 300 °C for 1 min. The AuNPs were then plasma cleaned (5 min, 300 W, Ar:O_2_ at 20:80%, 50 Pa), followed by the deposition of a 20 nm SiO_2_ layer via e-beam evaporation, which was subsequently thickened to approximately 300 nm using PECVD (40 min, ICP = 600 W, 400 Pa, SiH_4_:N_2_O 3:13 sccm). The SiO_2_/AuNP/Al structure was affixed to a 1.1 mm glass slide with epoxy. The aluminum oxide layer was scraped, and the sample was etched in an HCl/CuCl_2_ solution to transfer the AuNP layers. These layers were partially etched using reactive ion etching (RIE, 2 min, 100 W, Ar:CHF_3_:O_2_ at 30:15:5 sccm) to expose the AuNPs. The final sensor samples, sized at 9 × 9 mm^2^, were cut, washed with isopropyl alcohol, and dried with a nitrogen stream.

The sensing performances of single, double, and triple AuNP layers were evaluated by measuring their bulk refractive index sensitivity (RIS), determined from LSPR band shifts due to changes in the surrounding liquid’s refractive index [144]. Real-time transmittance spectra were obtained using a custom high-resolution LSPR spectroscopy system (Figure 8b). Figure 8c illustrates the morphology of the AuNP layers, showing oblate spheroid-shaped nanoparticles on SiO_2_ pillars, with diameter and thickness increasing from single to triple layers. Figure 8d depicts the proposed gas sensitivity functions (GS(t)) for each nanoparticle configuration during gas exchange tests.

It is apparent that the GS(t) functions for the double-particle configuration remained stable, regardless of the gas type being tested. Additionally, these gas sensitivity functions showed a strong correlation with the measured bulk RIS values [144].

### 4.4. Food Safety and Quality Control

Ensuring the safety and quality of food products is another critical application of SPR biosensors [145]. These devices detect allergens, contaminants, and spoilage organisms in food matrices, offering rapid and accurate testing that surpasses conventional methods [146,147]. For instance, SPR can identify trace levels of peanut proteins in processed foods, or monitor bacterial contamination in perishable items [148]. This real-time monitoring helps prevent foodborne illnesses and ensures compliance with safety standards, thereby protecting consumers and reducing economic losses in the food industry [146].

Podunavac et al. introduced a microwave microfluidic sensor utilizing spoof surface plasmon polaritons (SSPPs) for highly sensitive dielectric constant detection [149]. A novel SSPP unit cell was designed, with its behavior and sensing capabilities thoroughly examined. Using this unit cell, a multilayer SSPP microwave structure integrated with a microfluidic reservoir was developed as a sensing platform for liquid samples. Fabrication combines cost-effective methods, including xurography, laser micromachining, and cold lamination bonding. The sensor’s performance was validated experimentally using edible oil samples, showing high sensitivity (850 MHz per dielectric constant unit) and excellent linearity (R^2^ = 0.9802). Its affordability and straightforward fabrication make this sensor highly suitable for detecting minor dielectric constant variations in edible oils and other liquids [149].

**Figure 9:**
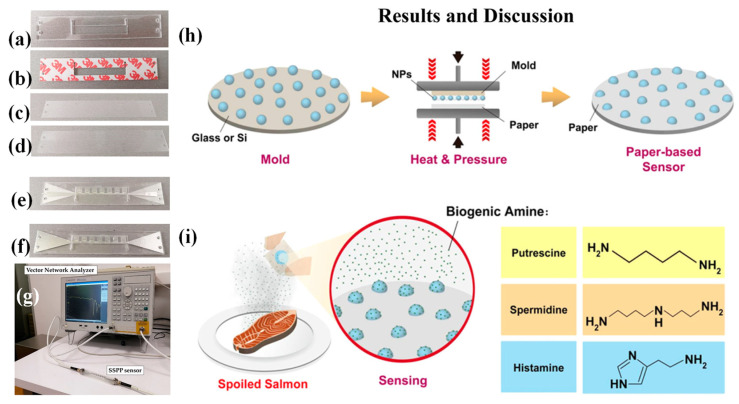
Components of the fabricated SSPP sensor: (**a**) PMMA layer featuring the microfluidic reservoir; (**b**) 3M double-sided adhesive tape [149]; (**c**) top layer constructed from PVC foil [149]; (**d**) bottom layer made of PVC foil; (**e**) top-view layout of the completed structure; (**f**) bottom-view layout of the completed structure [149], (**g**) measurement setup [149]. Illustrations depicting (**h**) the process of transferring metal NPs onto inkjet paper through imprinting [150] and (**i**) the application of NP-embedded paper as a gas sensor for detecting biogenic amine vapors emitted by spoiled food [150].

Figure 9a–f illustrates the design of each fabricated layer. The same pattern was employed for both the PMMA and 3M tape layers, as depicted in Figure 9a,b, respectively. The top PVC foil, shown in Figure 9c, includes inlet and outlet holes for sample injection into the microfluidic reservoir, while the bottom PVC foil, depicted in Figure 9d, serves to seal the channel system. The assembled sensor structure was displayed in Figure 9e,f, providing top and bottom views, respectively. The SSPP sensor was initially tested with palm oil, the sample with the lowest dielectric constant among those prepared. For each subsequent measurement, the reservoir was rinsed with the sample having the next highest dielectric constant before being filled with that sample. Due to the small volume of the microfluidic reservoir, only 0.8 mL of sample was required. Sensor responses were recorded using a vector network analyzer (VNA) E5071C from Agilent Technologies, with surface mount assembly (SMA) connectors (SMA Southwest Microwave 292-04A-5) facilitating the connection between the VNA and the SSPP sensor, as shown in Figure 9g. A single-point calibration was performed with the reservoir filled with air [149].

Tseng et al. developed a novel approach for paper-based plasmonic refractometric sensors by embedding metal nanoparticles (NPs) onto flexible paper substrates using a reversible nanoimprint lithography (NIL) technique [150]. These NP-integrated papers were designed as gas sensors for detecting volatile biogenic amines (BAs) emitted from spoiled food. The substrates used were commercial inkjet papers, known for their high reflectance (>80%) and smooth surfaces, with roughness around 4.9 nm. These properties made them suitable for reflection-mode plasmonic refractometric sensing, providing strong optical signals and efficient nanoparticle transfer. Additionally, the lightweight, flexible, and combustible nature of inkjet paper makes it ideal for creating portable, disposable, and environmentally friendly sensing platforms.

Solid silver nanoparticles (SNPs), gold nanoparticles (GNPs), and hollow Au–Ag alloy nanoparticles (HGNs) were first immobilized onto a solid mold and then transferred onto softened paper surfaces (Figure 9h). The density and exposure height of the embedded nanoparticles were influenced by imprinting parameters such as temperature and pressure. The optimal configurations achieved approximately 85% particle transfer efficiency, with around 50% of the particle surface area exposed, resulting in pronounced resonance reflectance dips for accurate detection. The HGN-embedded paper exhibited significant wavelength shifts when exposed to BA vapors, such as a Δλ of 33 nm for putrescine and 24 nm for spermidine, demonstrating high refractometric sensitivity. No notable spectral responses were observed for other gases like air, N_2_, CO_2_, or water vapor under typical food storage conditions, highlighting the sensor’s selectivity.

To assess the refractometric sensitivity of these LSPR-based paper sensors, volatile BAs were chosen as target analytes. The spectroscopic behavior of the NP-embedded paper upon BA adsorption was carefully analyzed (Figure 9i). BAs, small volatile organic bases commonly found in spoiled food, include putrescine, spermidine, histamine, cadaverine, tyramine, and spermine, all of which contain amino groups. During detection, these gaseous BAs bind to the NP surfaces through their amino groups, forming a stable adsorption layer via intermolecular interactions. This alters the local RI around the nanoparticles, causing a redshift in the LSPR wavelength. BAs are produced during food spoilage through the microbial decarboxylation of amino acids, a process driven by specific bacterial strains under improper storage or handling conditions. As microbial activity increases, so does the concentration of BAs, which can serve as reliable indicators of food quality and freshness. High levels of BAs also pose health risks and may contribute to foodborne illnesses [150].

### 4.5. Emerging Applications

SPR biosensors are expanding into emerging applications, driven by advancements in nanotechnology and artificial intelligence (AI) [151,152,153]. Integration with nanomaterials such as Au nanoparticles enhances the sensitivity and specificity of SPR sensors, enabling the ultra-sensitive detection of analytes at picomolar concentrations. AI algorithms further improve data analysis, enabling pattern recognition and predictive diagnostics [154]. These innovations are paving the way for wearable SPR-based sensors that provide real-time, continuous health monitoring and remote diagnostics [155,156]. Such devices hold immense potential for personalized healthcare and chronic disease management, especially in telemedicine settings [155,157,158]. Given the environmental risks to human health and the role of SPR in monitoring such issues, SPR has shown significant potential, particularly for detecting low-molecular-weight environmental contaminants in complex samples. Despite these benefits, challenges such as data analysis, sensor accuracy and reliability, and low signal-to-noise ratios remain. These challenges can be addressed with machine learning (ML), which can analyze extensive datasets, generate reliable outcomes even from noisy or low-resolution data, and identify connections between signals and biological events [159].

Ensuring the accuracy of responses from SPR sensors is crucial, particularly in applications such as substance detection, diagnostics, and routine testing. Mismanaged samples, instrumental noise, or molecular alterations can compromise the reliability of the data. Gomes et al. explored the application of machine learning (ML) techniques to address these challenges, enhancing the quality and dependability of real-time SPR sensorgram analysis [160]. A novel methodology for characterizing SPR sensorgrams was presented. The results demonstrate that the ML-based approach enabled the development of intelligent SPR sensors capable of providing secure, reliable, and auditable sensorgram evaluations. The proposed framework can be integrated into an Intelligence Module to classify sensorgrams and identify substances. It also facilitated the segmentation and analysis of key sensorgram regions and standardized data, and it supports audit processes. These advancements position next-generation SPR biodetection systems to perform automated and intelligent testing. The effectiveness of this system was validated using an SPR protocol designed for Leishmaniasis diagnosis, showcasing its potential for reliable and automated diagnostics [160].

Moreover, a photonic crystal fiber (PCF)-based biodetection system utilizing SPR was introduced for detecting malaria-infected red blood cells (RBCs) and hemoglobin (Hb) concentration [151]. The design incorporated a Ti_3_C_2_T_x_ thin film coated over a gold-layered PCF for SPR functionality. Malaria stages in RBCs were identified by comparing the resonance wavelengths of healthy and infected samples. Finite element method (FEM) simulations evaluated the sensor’s performance, yielding wavelength sensitivities of 12,142 nm/RIU for the ring stage, 9736 nm/RIU for the trophozoite stage, and 8241 nm/RIU for the schizont stage. Hb concentration detection achieved a maximum wavelength sensitivity of 53 nm/g/dL, with a resolution of 10^−5^ RIU. Additionally, the ML algorithm was applied, achieving a low mean squared error of 0.01526 and less than 2% error in sensitivity analysis. The proposed sensor’s enhanced performance and ML integration make it a promising alternative to existing malaria detection sensors.

Ehyaee et al. proposed a PCF sensor based on SPR with four Au nanowires to improve sensing performance [152]. ANN was used to predict confinement loss and sensitivity without requiring the imaginary part of the effective RI. The model showed reliability, with mean squared errors of 0.084, 0.002, and 0.003. The sensor achieved wavelength sensitivities of 2000–18,000 nm/RIU for refractive indices of 1.31–1.4 (720–1280 nm range) and a maximum amplitude sensitivity of 889.89 RIU^−1^. This integration of SPR, photonic crystal fiber, and ML enhanced sensor performance and offered an efficient predictive methodology, highlighting the potential of ML in advancing optical sensor technologies [152].

**Figure 10:**
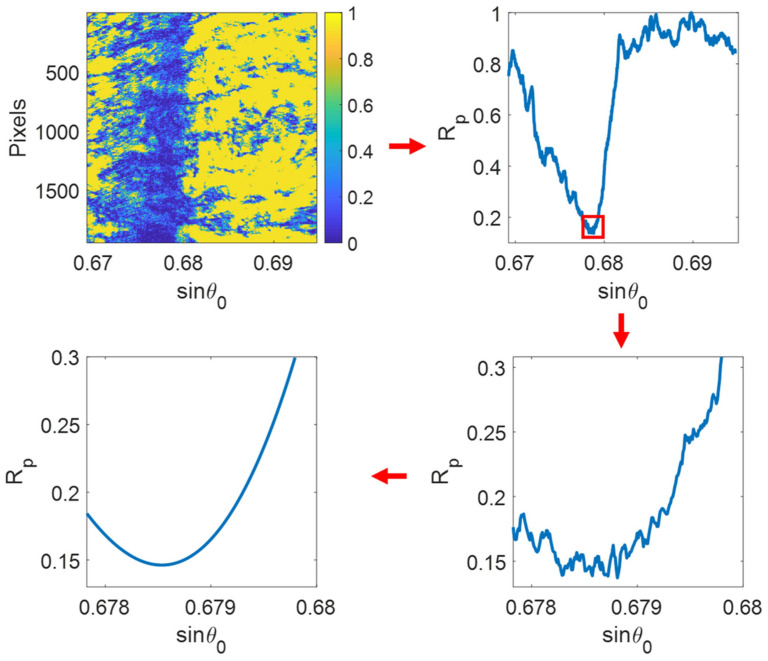
The steps involved in determining the minimum position of the SPR reflectance dip using the cubic polynomial curve fitting method [161].

Angular scanning-based SPR measurement is widely employed in label-free sensing applications. The accuracy and precision of these measurements are strongly dependent on the precise determination of the plasmonic angle. Various techniques have been introduced in the literature to achieve this, including polynomial curve fitting, image processing approaches, and image averaging. For intensity detection, the achievable precision for SPR is approximately within the range of 10^−5^ RIU to 10^−6^ RIU. Thadson et al. introduced a deep learning (DL) approach for plasmonic angle detection, aimed at improving accuracy without requiring advanced post-processing, specialized optical setups, or traditional polynomial curve fitting techniques [161]. The proposed method leveraged a straightforward convolutional neural network (CNN) architecture trained on simulated reflectance spectra. These spectra incorporated shot noise and speckle noise to enhance the generalizability of the training dataset. Validation of the network was performed using an experimental setup to measure refractive indices of air and nitrogen gas at varying concentrations. The precision obtained from experimental reflectance images using the proposed method is 4.23 × 10^−6^ RIU, surpassing the cubic polynomial curve fitting precision of 7.03 × 10^−6^ RIU and the 2D contour fitting precision of 5.59 × 10^−6^ RIU achieved with Horner’s method. The process flows for determining the plasmonic angle from a recorded camera frame are illustrated in Figure 10. The initial step involved preparing the line-scan reflectance by averaging all rows in the camera frame. A cubic polynomial curve was then applied through the minimum reflectance of the averaged SPR dip. The accuracy of this curve fitting method was influenced by the number of data points used in the polynomial fitting [161].

**Table 1:** SPR-based sensors across a wide range of fields.

Application	Key Features	Examples of Detection Targets	Benefits
Biomedical Diagnostics	High sensitivity, real-time monitoring, label-free detection [162]	Biomolecules (e.g., proteins, DNA, antibodies), disease biomarkers [163,164]	Rapid diagnosis, early detection, personalized medicine [165,166]
Pharmaceutical and Drug Discovery	High-throughput screening, kinetic studies, quantitative binding analysis	Drug–target interactions, ligand–receptor binding, enzyme activity	Accelerates drug discovery, precise kinetic profiling, reduced reagent consumption
Environmental Monitoring	Detection of pollutants, toxins, and pathogens in water, air, and soil	Heavy metals, pesticides, pathogens, harmful gases	Real-time monitoring, early warning systems, high specificity
Food Safety and Quality Control	Assessment of contaminants, pathogens, and adulterants [167]	Foodborne pathogens (e.g., *E. coli*, *Salmonella*), toxins, allergens [168]	Ensures food safety, compliance with regulations, non-destructive testing [169,170,171,172]
Emerging Applications	Innovations in wearable sensors, remote monitoring, and integration with IoT	Continuous glucose monitoring, pathogen detection in smart packaging	Versatility, integration with advanced technologies, enhanced accessibility and convenience

## 5. Advancement in SPR Technology

Advancements in SPR technology have broadened its applications and capabilities, driving innovations in biosensing and chemical analysis. From the integration of nanostructures to the development of hybrid systems and portable devices, these advancements are paving the way for more efficient, sensitive, and versatile sensing platforms.

### 5.1. LSPR

LSPR harnesses the unique optical properties of nanostructures and plasmonic nanoparticles, such as Au and Al, to enhance sensor performance [173,174]. The confinement of plasmonic resonances at the nanoparticle scale enables LSPR-based sensors to achieve higher sensitivity compared to traditional SPR systems [175]. The tunable optical properties of nanostructures, achieved through variations in size, shape, and material composition, allow precise control over the resonance wavelength and field enhancement [176,177]. LSPR sensors also offer remarkable miniaturization potential, making them suitable for integration into compact diagnostic tools [175]. Their small sensing volume and localized fields make them ideal for detecting low-abundance analytes with high specificity. These features have enabled breakthroughs in biomedical diagnostics, environmental monitoring, and drug discovery, where sensitivity and device portability are critical [178,179,180,181].

Advancements in technology continue to enable innovative approaches to cost-effective and practical biosensing solutions. Islam et al. introduced an LSPR system that integrated wave-guiding and plasmonic resonance sensing within a single microstructured polymeric device. FEM simulations used for sensor characterization revealed an unprecedented wavelength sensitivity of 111,000 nm/RIU, alongside a high amplitude sensitivity of 2050 RIU^−1^ [182]. The sensor also achieved remarkable resolution and LODs of 9 × 10^−7^ RIU and 8.12 × 10^−12^ RIU^2^/nm, respectively. Additionally, it can detect analytes across an RI range of 1.33–1.43, covering the visible to mid-IR spectrum. These characteristics make it a promising candidate for detecting biomolecular and chemical analytes [182].

Lugongolo et al. investigated the effectiveness of an LSPR biodetection system in identifying a single nucleotide mismatch in DNA sequences [183]. The detection mechanism relied on the hybridization of a 100 ngμL^−1^ target DNA with two biotinylated probes: one fully complementary and the other partially complementary with a single nucleotide mismatch, both applied at 0.1 μm concentrations on a Au-coated surface. The LSPR biodetection system demonstrated sensitivity by distinguishing sample M+ from sample C+ through transmission intensity variations of 0.28 and 0.26 μA, respectively. These results highlight the sensor’s ability to differentiate single-base-pair differences, presenting a promising avenue for developing point-of-care devices. This simplified and cost-effective method holds the potential for detecting biologically and clinically significant mutations, including those linked to antimicrobial resistance. Ongoing research aims to further evaluate the robustness of the LSPR biodetection system using the biotin–neutravidin technique [183].

**Figure 11:**
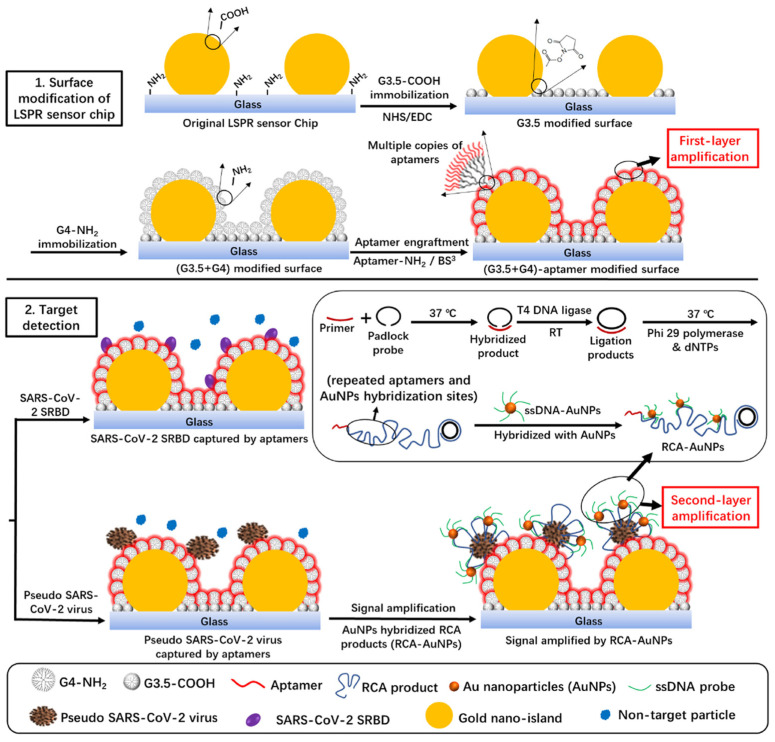
A schematic illustration outlines the process of preparing (G3.5 + G4)-aptamer-modified LSPR sensor chips for detecting the SARS-CoV-2 SRBD and pseudo viral particles. The second-layer amplification is applicable only when detecting SARS-CoV-2 pseudo viral particles, enabling the use of a detection sandwich format [175].

Hao et al. devised a comprehensive strategy to enhance the detection sensitivity of LSPR sensor chips for SARS-CoV-2 detection [175]. Poly(amidoamine) dendrimers were immobilized on the LSPR sensor chip surfaces, serving as scaffolds for conjugating aptamers specific to SARS-CoV-2. This modification minimized nonspecific surface adsorption and increased the density of capturing ligands, thereby improving sensor sensitivity. To evaluate the performance of the modified chips, the SARS-CoV-2 spike protein receptor-binding domain was tested using LSPR chips with varying surface modifications [175]. The dendrimer-aptamer functionalized chips achieved an LOD of 21.9 pM, demonstrating sensitivity improvements of 9-fold and 152-fold over conventional aptamer- and antibody-based LSPR chips, respectively. Furthermore, sensitivity was significantly enhanced by integrating rolling circle amplification products and Au nanoparticles, which amplified detection signals by increasing target mass and plasmonic coupling effects (Figure 11). Tests with pseudo-SARS-CoV-2 viral particles confirmed that this approach improved sensitivity by 10-fold, achieving an exceptional LOD of 148 vp/mL. This represents one of the most sensitive SARS-CoV-2 detection methods reported, underscoring the potential of this advanced LSPR platform for the rapid and highly sensitive detection of COVID-19 and other viral infections in point-of-care settings.

### 5.2. Hybrid and Multi-Modal Systems

The fusion of SPR with other sensing modalities has led to hybrid systems that capitalize on complementary detection principles, offering improved versatility and accuracy [184,185]. Integrating SPR with electrochemical sensors allows simultaneous optical and electrical signal readouts, enhancing detection reliability [186,187]. Similarly, coupling SPR with fluorescence techniques amplifies signal outputs, enabling the detection of ultra-low concentrations of analytes [188]. Photonic crystals and metasurfaces play a pivotal role in these hybrid systems by enhancing light–matter interactions. These engineered materials optimize resonance conditions, improve signal quality, and provide additional tunability for sensor applications [189,190].

Wakalao et al. introduced the theoretical modeling and design of an advanced metasurface-based sensor aimed at cervical cancer detection [189]. The sensor leveraged graphene, black phosphorus, and titanium dioxide as its key sensing components. It operated in dual bands (1.369–1.383 THz and 0.313–0.317 THz) and delivered outstanding performance, including a sensitivity of 400 GHz/RIU, a figure of merit of 5.882 RIU^−1^, and Q-factors ranging from 9.206 to 9.950. The sensor’s dual-band capability, along with its 2-bit encoding features, highlighted its potential for use in multi-parameter analysis and advanced information processing, enabling more comprehensive diagnostics. Additionally, integrating Support Vector Regression (SVR) with a polynomial kernel demonstrated exceptional efficiency, achieving a perfect R^2^ score of 100%, while reducing simulation time by 80% and significantly lowering the computational effort needed for sensor optimization [189]. Hybrid systems have opened up new possibilities for multi-analyte detection on a single platform, particularly in complex biological and environmental samples [185,191].

SPR and Love wave (LW) surface acoustic wave (SAW) sensors are widely recognized for their reliability in the real-time, label-free detection of biomolecular interactions. Samarentsis et al. developed an integrated platform combining SPR and LW-SAW technologies to enable the simultaneous optical and acoustic analysis of biomolecular binding on a shared surface [192]. This system measured two acoustic parameters—the phase and amplitude of the LW—alongside SPR data. A unique 3D-printed plastic holder and a PDMS microfluidic cell were incorporated into the experimental setup, supporting a flow-through operation. The platform was systematically evaluated using various surface modifications, such as rigid mass loading (via Au deposition), viscous loading (using glycerol and sucrose solutions), and protein adsorption (BSA), to study both optical and acoustic responses.

**Figure 12:**
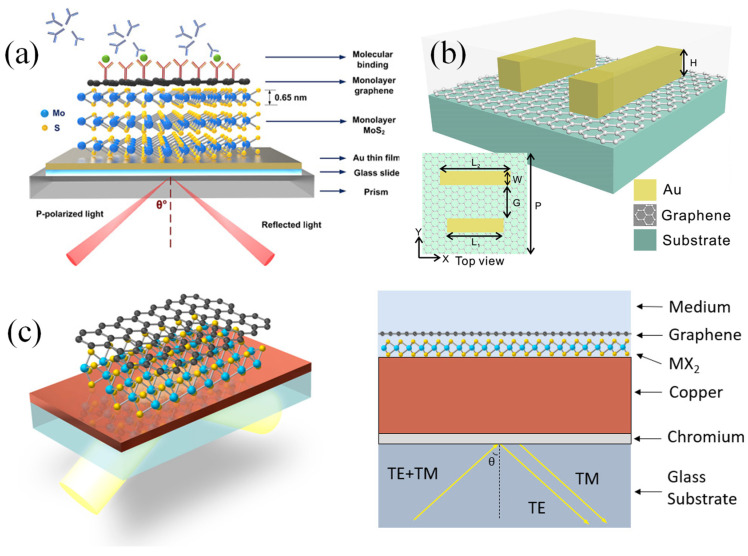
(**a**) Illustration of the graphene–MoS_2_-enhanced SPR biodetection system. (**b**) Schematic of linearly polarized waves (x-polarization) incident normally on a Au nano-antenna/graphene hybrid structure in a Cartesian coordinate system. The multilayer structure includes a cover layer, a periodic array of asymmetric Au nano-antennas, an unpatterned graphene monolayer, and a semi-infinite substrate [194]. (**c**) Schematic of the Cu–TMDCs–graphene-enhanced SPR biodetection system. The GH shift difference between TM and TE waves was measured to improve the signal-to-noise ratio, using TE wave signals as a reference [188].

Zeng et al. proposed an advanced SPR sensor design incorporating graphene–MoS_2_ hybrid structures for highly sensitive molecular detection (Figure 12a) [193]. This configuration demonstrated phase-sensitivity improvements exceeding 500 times compared to conventional SPR systems with either no graphene–MoS_2_ layers or graphene alone. The enhancement was attributed to monolayer MoS_2_’s superior optical absorption efficiency (~5%) compared to graphene’s (~2.3%). Analysis indicated that the electron energy loss in MoS_2_ was comparable to that of graphene, enabling nearly complete (~100%) light energy transfer to the graphene–MoS_2_-coated substrate, significantly amplifying SPR signals. Simulations showed that the design produced a quasi-dark reflected light point, leading to a pronounced phase shift at the resonance angle. The phase interrogation method applied in this system achieved greater sensitivity than traditional angular interrogation techniques. Theoretical studies identified optimal design parameters, including a 45 nm-thick Au film and three layers of MoS_2_ nanosheets, to maximize detection sensitivity [193].

Hong et al. developed a hybrid plasmonic sensor that integrated metal and graphene components for multi-spectral sensing in both the NIR and MIR spectral ranges [194]. The sensor’s design incorporated an array of asymmetric Au nano-antennas combined with a continuous graphene sheet (Figure 12b). The Au nano-antennas produced distinct Fano resonances for NIR sensing, while the graphene plasmonic resonances extended the sensor’s functionality into the MIR range, providing a wider spectral range compared to earlier multi-spectral sensors. The sensitivity and FOM of the sensor were comprehensively evaluated, examining how these parameters were influenced by the thickness of the sensing layer and the Fermi energy of graphene. By merging the advantages of traditional metal-based plasmonic sensors with graphene’s unique properties, this design introduced a versatile platform for advanced multi-functional plasmonic sensing applications [194].

Zakirov et al. introduced an SPR biodetection system design utilizing a copper nanosubstrate integrated with graphene and 2D transition metal dichalcogenides (TMDCs) for ultrasensitive detection [188]. The system comprised seven layers, as follows: an SF11 triangular prism, BK-7 glass, a chromium adhesion layer, a thin copper film, a TMDC layer (MoS_2_, MoSe_2_, WS_2_, or WSe_2_), graphene, and a sensing layer containing biomolecular analytes (Figure 12c). Copper was selected as the plasmonic material for its superior conductivity compared to Au, cost-effectiveness, and scalability. Sensitivity calculations were performed using the Goos–Hänchen (GH) shift method, which measured the lateral displacement of the p-polarized reflected beam under total internal reflection, derived from phase changes. The GH-based SPR signal was significantly more sensitive than intensity-based methods, such as angular or wavelength scanning, due to the steep phase variation of the reflected light. By optimizing copper thickness, the number of 2D material layers, and excitation wavelength, the design achieved enhanced sensitivity, with an LOD of 10^−9^ RIU [188].

### 5.3. Portable and Wearable SPR Devices

The development of portable and wearable SPR devices represents a significant leap toward practical, on-the-go sensing applications [181,195]. Advances in microfabrication techniques have enabled the creation of compact SPR systems with integrated optics and microfluidics, reducing the size and cost of traditional SPR instruments. Wireless technologies further enhance these systems, allowing real-time data transmission and remote monitoring [196,197]. Examples of field-deployable SPR devices include smartphone-integrated sensors for rapid diagnostics, handheld devices for environmental analysis, and wearable biodetection systems for continuous health monitoring.

A life-threatening anaphylactic shock can occur if IgA-containing blood is administered to a patient with undiagnosed immunoglobulin A (IgA) deficiency (defined as IgA levels < 500 ng/mL), emphasizing the need for a rapid, point-of-care (POC) method for IgA deficiency screening. While an enzyme-linked immunosorbent assay (ELISA) is commonly used to detect IgA, this technique requires trained specialists and at least 24 h to provide results. To address this limitation, an SPR-based protocol has been developed to identify IgA-deficient patients or donors within 1 h [198]. These innovations are transforming SPR technology into a user-friendly tool for non-expert operators, promoting its adoption in diverse fields such as healthcare, agriculture, and public safety [34,199].

**Figure 13:**
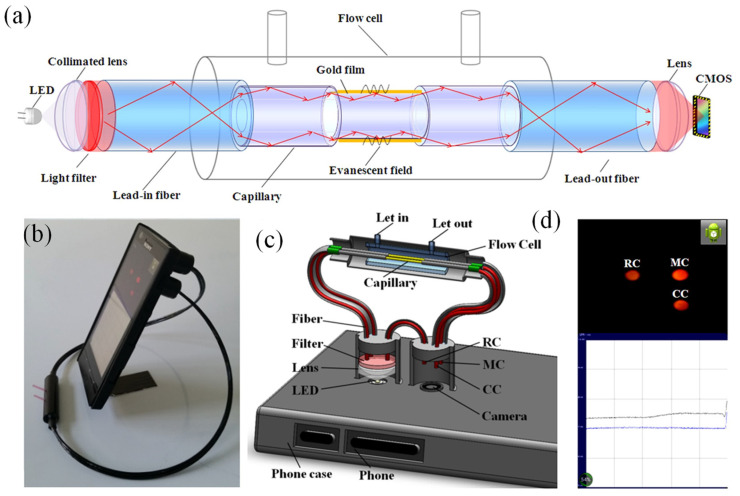
Instrumentation of the smartphone-based SPR imaging biosensor: (**a**) Diagram depicting the structure of the smartphone-based SPR sensor. (**b**) Photograph showing the SPR sensor mounted on an Android smartphone. (**c**) Three-dimensional illustration detailing the internal configuration of the opto-mechanical attachment. (**d**) The smartphone camera captures images of the measurement, control, and reference channels, which are quickly analyzed to determine relative intensity. The results are plotted and displayed on the smartphone screen [199].

Liu et al. presented a fiber optic SPR biosensor designed for integration with smartphone platforms [199]. The system comprises lightweight optical components and a sensing element interconnected via optical fibers mounted on a phone case. Figure 13a–d illustrates a schematic, photograph, and interface of the detection system. The system’s components were mounted on the phone case without obstructing the touch screen or display during use. This design allowed the smartphone and sensing components to function as a compact instrument that can be easily assembled or disassembled. Before mounting, the ends of the lead-in and lead-out fibers (hard plastic cladding silica optical fibers) were polished for optimal alignment with the phone’s camera and LED flash. These fibers were secured in designated slots on the case, ensuring proper positioning. A low-cost plastic lens collimated the red light emitted by the LED flash, which acts as a cold light source. To reduce stray light, the fibers were enclosed in black rubber tubing. The fibers were connected to the sensing elements via optical fiber connectors, enabling the easy integration and replacement of components. The system compensated for the LED flash’s power instability using a reference channel alongside the measurement and control channels. The reference fiber was positioned adjacent to the lead-in fibers to ensure consistent light conditions, mitigating fluctuations in light intensity for reliable biosensing. This cost-efficient and portable smartphone-based SPR biodetection system offers significant potential for applications in fields like healthcare, medical diagnostics, and environmental monitoring [199].

## 6. Challenges and Future Perspectives

The utilization of SPR technology, while transformative in various analytical and diagnostic applications, still faces notable limitations that impact its broader adoption. Key among these are challenges related to sensitivity, specificity, and stability [200]. Sensitivity issues arise when detecting low-abundance analytes in complex biological matrices, where background noise and interference from non-specific binding can obscure meaningful signals [201]. Specificity is another critical concern; the accuracy of SPR relies heavily on the interaction between the immobilized recognition element (e.g., antibodies or aptamers) and the target analyte [202]. Any cross-reactivity or degradation of the recognition element can significantly impair performance [203]. Stability is also a pressing issue, as the reproducibility of results is contingent on the physical and chemical robustness of the SPR sensing layer and associated components, which may degrade over time or under adverse environmental conditions [204].

Additionally, the cost and complexity of fabrication remain significant barriers to widespread SPR implementation. The construction of SPR sensors involves precise manufacturing techniques and high-quality materials, particularly for the Au or Ag layers used to sustain plasmonic resonances. Integrating these components into a reliable device often necessitates expensive equipment and highly skilled personnel, driving up the overall cost. The need for precise control over microfluidic channels and coupling optics further contributes to the complexity of fabrication, making it challenging to scale production for cost-effective, high-volume use. These limitations underscore the necessity for continued innovation to refine SPR systems and broaden their accessibility [205].

Emerging trends in SPR technology are paving the way for enhanced functionality and broader applications. One of the most promising developments is the integration of artificial intelligence (AI) and machine learning (ML) into SPR data analysis [206,207]. SPR generates complex datasets that often require advanced interpretation to extract meaningful insights, particularly in high-throughput or multiplexed setups [208]. AI and ML algorithms can automate the analysis process, identifying subtle patterns in data and improving both sensitivity and specificity [152,209]. For example, ML models can be trained to distinguish between signal variations caused by genuine analyte binding and those resulting from noise or non-specific interactions. These technologies not only accelerate data processing but also open up new possibilities for real-time monitoring and decision-making in clinical and research settings.

**Figure 14:**
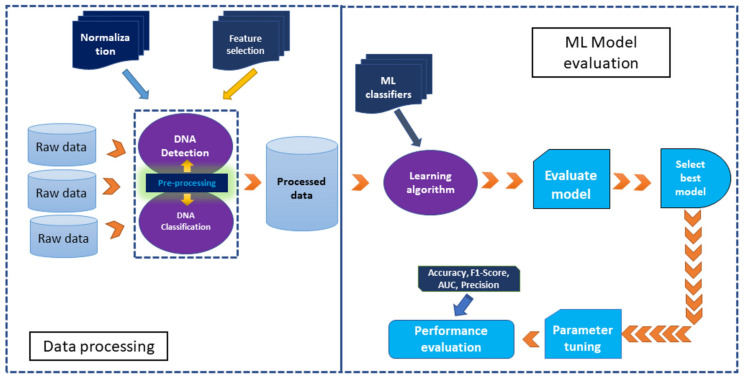
Flowchart of the ML models [210].

Mondal et al. introduced innovative ML-driven approaches for DNA detection and classification using reflective light angles and related properties of Au surfaces in SPR biodetection systems [210]. The flow chart of the ML model’s development is shown in Figure 14. Extensive statistical analyses and visualization techniques were employed to assess the dataset, with t-SNE feature extraction and min–max normalization applied to enhance classifier differentiation, especially for low-variance features. The experiments involved various ML classifiers, including support vector machine (SVM), decision tree (DT), multi-layer perceptron (MLP), k-nearest neighbors (KNN), logistic regression (LR), and random forest (RF). Performance was evaluated using metrics such as accuracy, precision, F1-score, and area under the receiver operating characteristic curve (AUC). The results indicate that RF, DT, and KNN achieved the highest accuracy of 0.94 for DNA classification, while RF and KNN reached 0.96 accuracy in DNA detection tasks. RF also demonstrated superior performance across AUC (0.97), precision (0.96), and F1-score (0.97) for both tasks. These findings underscore the potential of ML models to enhance biosensor development, paving the way for innovative tools in disease diagnosis and prognosis [210].

Another significant trend is the advancement of SPR materials and device integration [211]. Researchers are exploring novel plasmonic materials, such as graphene, black phosphorus, and various nanocomposites, to replace or complement traditional Au and Ag layers [212]. These materials offer enhanced optical properties, greater chemical stability, and improved sensitivity, broadening the range of detectable analytes. In parallel, efforts to miniaturize SPR devices and integrate them with complementary technologies, such as microfluidics and lab-on-a-chip platforms, are making the technology more versatile and portable. These advancements are particularly crucial for applications in point-of-care diagnostics, where compact and user-friendly devices are essential.

Looking ahead, SPR technology holds immense potential to contribute to emerging scientific and medical fields. One exciting frontier is its application in synthetic biology and quantum biosensing. In synthetic biology, SPR can be employed to monitor molecular interactions and engineer custom biological systems with unprecedented precision. The technology’s ability to provide real-time, label-free analysis makes it ideal for characterizing synthetic proteins, nucleic acids, and other biomolecules. In quantum biosensing, SPR could play a role in harnessing quantum effects for the ultra-sensitive detection of biological markers, potentially revolutionizing fields such as early cancer detection and single-molecule diagnostics [213].

Another promising opportunity lies in the expansion of SPR technology into low-resource settings and global health initiatives. By developing cost-effective and robust SPR systems, the technology could be adapted for use in areas with limited access to advanced healthcare infrastructure. Such systems could facilitate rapid diagnostics for infectious diseases, water quality monitoring, and agricultural pathogen detection, addressing critical challenges in global health and sustainability. Innovations in device portability, affordability, and ease of use will be essential to realizing this vision, ensuring that the benefits of SPR reach underserved populations worldwide [214]. Together, these opportunities highlight SPR’s transformative potential across diverse disciplines, positioning it as a cornerstone technology for addressing some of the most pressing challenges in science, medicine, and global health [215].

## 7. Conclusions

SPR-based biodetection systems have established themselves as a pivotal technology within analytical and diagnostic sciences, characterized by their exceptional sensitivity and adaptability. Over the past few decades, these biodetection systems have evolved from fundamental research instruments into extensively utilized platforms, driven by significant advancements in nanotechnology, materials science, and computational data analytics. Modern SPR systems are distinguished by their capacity for real-time, label-free detection with extraordinary sensitivity, enabling comprehensive applications in biomolecular interaction analysis, clinical diagnostics, environmental surveillance, and pharmaceutical development.

Recent advancements in SPR technology have addressed critical challenges associated with sensitivity enhancement, specificity, and device miniaturization. The incorporation of nanostructures such as Au nanoparticles and metamaterials has substantially amplified plasmonic resonance, achieving LODs in the picomolar to femtomolar range. Moreover, progress in surface functionalization methodologies, including the deployment of aptamers and molecularly imprinted polymers, has greatly enhanced molecular recognition specificity. Additionally, integration with complementary technologies such as microfluidics and artificial intelligence has enabled high-throughput capabilities and optimized analytical performance, further broadening the potential applications of SPR biosensors.

The transformative potential of SPR-based biodetection systems across diverse scientific and industrial sectors is undeniable. In medical diagnostics, these biosensors are set to revolutionize personalized medicine through the early and accurate detection of diseases, real-time therapeutic monitoring, and drug efficacy evaluation. In environmental science, SPR biodetection systems provide robust and precise tools for detecting environmental contaminants and pathogens within complex matrices. Their industrial utility is equally significant, with impactful applications in food safety assurance, bioprocess monitoring, and quality control. Furthermore, the simplicity, scalability, and reusability of SPR platforms enhance their suitability for deployment in resource-constrained settings, promoting global accessibility.

The ongoing development of SPR technology is expected to drive groundbreaking advancements in multidisciplinary research and industrial practices. Innovations in plasmonic materials, advanced signal processing, and sensor miniaturization are likely to further expand the versatility and efficiency of SPR-based systems, solidifying their role as a cornerstone of modern science and technology. As SPR biosensors continue to bridge the gap between basic research and practical implementation, their societal impact will be profound, heralding a future where real-time, precise, and cost-effective sensing solutions become integral to scientific and industrial processes.

## Data Availability

No new data were produced.
